# Green tea–derived exosome-like nanoparticles attenuate oxidative stress–induced skin senescence via modulation of p38 MAPK signaling

**DOI:** 10.3389/fphar.2026.1806328

**Published:** 2026-04-28

**Authors:** Wooram Choi, Jeong Hun Cho, Sang Hee Park, Dong Seon Kim, Hwa Pyoung Lee, Yong Deog Hong, Hyoung-June Kim, Byong Chul Yoo, Jongsung Lee, Jae Youl Cho

**Affiliations:** 1 Department of Integrative Biotechnology, Sungkyunkwan University, Suwon, Republic of Korea; 2 Research and Innovation Center, AMOREPACIFIC, Yongin, Republic of Korea; 3 Department of Biocosmetics, Sungkyunkwan University, Suwon, Republic of Korea; 4 InnoBation Bio, Seoul, Republic of Korea

**Keywords:** caffeine, cellular senescence, oxidative stress, p38 MAPK, plant-derived exosome-like nanoparticles, *Camellia sinensis* (L.) Kuntze [Theaceae]

## Abstract

**Introduction:**

Plant-derived exosome-like nanoparticles (PDENs) have emerged as bioactive nanostructures capable of transferring molecular cargos across species; however, their mechanistic roles in mammalian stress-responsive signaling remain incompletely defined. In this study, we investigated the effects of green tea (*Camellia sinensis* (L.) Kuntze [Theaceae])–derived exosome-like nanoparticles (GtDENs) on oxidative stress–induced cellular dysfunction in human keratinocytes.

**Methods:**

To do this, we employed MTT assay, immunoblotting analysis, real-time PCR method, SA-beta-gal assay, flowcytometry, and molecular docking model.

**Results and Discussion:**

GtDENs exhibited no detectable cytotoxicity and significantly attenuated hydrogen peroxide– and UVB-induced reactive oxygen species (ROS) accumulation and cell death. GtDENs suppressed oxidative stress–associated cellular senescence and apoptotic gene expression, including MMP3, p21, and caspases. Mechanistically, GtDENs preferentially reduced phosphorylation of p38 MAPK and downstream c-Fos activation under oxidative stress conditions, while ERK and JNK signaling remained largely unaffected. Functional analyses, including p38 overexpression and pharmacological inhibition experiments, support the involvement of p38 MAPK signaling in mediating GtDEN activity. Phytochemical profiling identified caffeine as the predominant small-molecule component within GtDENs. Molecular docking and cellular thermal shift assays suggest potential interaction between caffeine and p38 MAPK, although direct biochemical binding validation was not performed. Exploratory small RNA profiling further indicated the presence of regulatory RNA species associated with stress-responsive pathways. Collectively, these findings indicate that GtDENs modulate oxidative stress–responsive signaling, with p38 MAPK emerging as a key signaling axis involved in this response.

## 1 Introduction

Extracellular vesicles (EVs) have emerged as critical mediators of intercellular communication, enabling the transfer of bioactive molecules that regulate diverse physiological and pathological processes (Dang et al., 2020). EVs are broadly classified into microvesicles, apoptotic bodies, and exosomes based on their biogenesis and size (Dang et al., 2020; Kakarla et al., 2020; Ratajczak and Ratajczak, 2020). Among these, exosomes are nanoscale vesicles (20–100 nm) generated through the endosomal pathway and released upon fusion of multivesicular bodies with the plasma membrane (Samanta et al., 2018). Exosomes carry a complex molecular cargo, including nucleic acids, proteins, and metabolites, and are enriched in tetraspanins such as CD9, CD63, and CD81, which are commonly used as molecular markers (Gao et al., 2021). Through these cargos, exosomes function as biologically active delivery vehicles that modulate signaling pathways in recipient cells (Maia et al., 2018).

In addition to mammalian systems, plants also release extracellular vesicle–like nanoparticles into the extracellular space in response to biotic and abiotic stress (Cai et al., 2019). These vesicles, commonly referred to as plant-derived exosome-like nanoparticles (PDENs) or plant-derived extracellular vesicles (PDEVs), share structural and functional similarities with mammalian exosomes (Regente et al., 2012). While increasing attention has been paid to the biogenesis and molecular composition of PDENs, their pharmacological relevance and mechanistic actions in mammalian cells remain incompletely understood, particularly in the context of stress-responsive signaling pathways.

Green tea, derived from the leaves of *Camellia sinensis*, is widely recognized for its health-promoting properties. Its bioactive constituents—including catechins, flavonoids, phenolic acids, and other metabolites—have been reported to exhibit antioxidant, anticancer, antiviral, and antibacterial activities (Zhao et al., 2022). Despite extensive studies on soluble green tea components, little is known about the biological functions of green tea–derived exosome-like nanoparticles (GtDENs) and their potential role as nanoscale carriers of bioactive molecules. Elucidating the signaling pathways targeted by GtDENs may provide mechanistic insight into their pharmacological actions.

Reactive oxygen species (ROS) are highly reactive molecules that play a dual role in cellular physiology, acting as signaling mediators under basal conditions but inducing cellular damage when excessively accumulated (Juan et al., 2021). Endogenously, ROS are generated primarily by mitochondria and chloroplasts during respiration and photosynthesis, respectively (Han et al., 2001), whereas exogenous sources include ultraviolet B (UVB) irradiation, heavy metals, tobacco smoke, and ionizing radiation (Muthukumar and Nachiappan, 2010). In human skin, UVB represents a major environmental source of ROS and is directly implicated in oxidative damage and premature aging (Li et al., 2024). In experimental systems, chemical oxidants such as hydrogen peroxide (H_2_O_2_) and tert-butyl hydroperoxide (t-BHP) are commonly used to induce intracellular oxidative stress (Nishi et al., 2017). Excessive ROS damage macromolecules, including DNA, and promote oxidative stress–driven cellular senescence (Cavazzoni et al., 1999; Valverde et al., 2018), highlighting the importance of pharmacological strategies that attenuate ROS-mediated signaling cascades.

One of the principal pathways activated by oxidative stress is the activator protein-1 (AP-1) signaling axis (Wu et al., 2005). AP-1 is a transcription factor complex composed mainly of c-Jun and c-Fos family proteins, whose activity is regulated by phosphorylation-dependent activation and nuclear translocation (Zenz et al., 2008). Activated AP-1 induces the transcription of matrix metalloproteinases (MMPs), leading to extracellular matrix degradation and contributing to skin aging (Benbow and Brinckerhoff, 1997; Jabłońska-Trypuć et al., 2016). In addition, AP-1 regulates the expression of genes involved in apoptotic and stress-response pathways (Jacobs-Helber et al., 1998). Accordingly, pharmacological inhibition of AP-1 signaling has been explored as a strategy to mitigate oxidative stress–induced aging and cellular dysfunction (Song et al., 2022).

The p38 mitogen-activated protein kinase (MAPK14) is a central upstream regulator of AP-1 signaling in response to oxidative and environmental stress (Slomiany and Slomiany, 2013). p38 is activated through dual phosphorylation at Thr180 and Tyr182 residues following exposure to stimuli such as UVB irradiation and H_2_O_2_ (Harada et al., 2014). Sustained p38 activation is closely associated with cellular senescence, growth arrest, and stress-induced functional decline in normal human cells (Iwasa et al., 2003). Thus, p38 MAPK represents a pharmacologically relevant target for modulating oxidative stress–driven signaling networks.

In the present study, we investigated the protective effects of green tea–derived exosome-like nanoparticles in human keratinocytes under oxidative stress conditions. We provide evidence that GtDENs attenuate ROS-induced cellular senescence and apoptotic signaling by modulating the p38 MAPK–AP-1 axis, providing mechanistic insight into the pharmacological actions of plant-derived exosome-like nanoparticles.

## 2 Materials and methods

### 2.1 Materials

HaCaT keratinocyte cell lines were obtained from Antibody Research Corporation (MO, United States). HEK293T cell lines were purchased from American Type Culture Collection (Rockville, MD, United States). The cell culture media DMEM (Dulbecco’s Modified Eagle Medium) and antibiotics (penicillin and streptomycin) were obtained from Hyclone (Logan, UT, United States). H_2_DCFDA was obtained from Thermo Fisher Scientific (Waltham, Massachusetts, United States). H_2_O_2_ and MTT were purchased from Sigma-Aldrich. All the primers used in this research were purchased from Macrogen (Seoul, Korea). The cDNA synthesis kit was produced by Thermo Fisher Scientific (Waltham, MA, United States). The antibodies for p-c-Jun (Ser73), c-Jun, p-c-Fos (Ser32), c-Fos, p-ERK (Thr202/Tyr204), ERK, p-JNK (Thr183/Tyr185), JNK, p-p38 (Thr180/Tyr182), p38, p-MKK3/6 (Ser189/207), and MKK3/6 were obtained from Cell Signaling Technology (Beverly, MA, United States). The antibody for β-actin was obtained from Santa Cruz Biotechnology, Inc. (Dallas, TX, United States).

### 2.2 Extraction and separation of GtDENs

Green tea leaves cultivated in Republic of Korea were supplied through Osulloc Farm Corporation. The fresh green tea leaves collected in September were washed twice with tap water, and after drying only on the surface, selected without defects. The plant materials were stored in −80 °C until the GtDENs extraction process. At the time of extraction, green tea leaves were thawed and GtDENs were obtained using a modified aqueous two-phase system–based protocol. In brief, green tea leaves (300 g) were soaked in 1 L deionized water (DW) and treated with 200 MPa pressure at 25 °C for 30 min. Then, the sample was ground and mixed with a juice mixer and the suspended debris was removed using a mesh filter. Aqueous two-phase extraction was performed to separate the GtDENs: 10,000–35,000 Da of polyethyleneglycol and 300,000–650,000 Da of dextran were added to the juice to reach the concentrations 3.3% and 1.7%, respectively. Centrifugation was conducted at 1,000 × *g*, 4 °C for 10 min to obtain the lower layer containing GtDENs. As a result, GtDENs were obtained in yielding ∼6 mL per batch for experiments. The isolation protocol used in this study was identical to that described in our previous report (Cho et al., 2022), in which cryo-transmission electron microscopy (cryo-TEM) confirmed the presence of lipid bilayer–enclosed vesicular structures. Although additional ultrastructural validation was not performed in the present study, the same extraction and purification procedure was applied to ensure methodological consistency. It should be noted that plant-derived extracellular vesicle isolation remains technically challenging, and the possibility of co-isolated soluble phytochemicals cannot be completely excluded.

### 2.3 Analysis of GtDENs with NTA

To verify the particle size and concentration, Zetaview system (Malvern Panalytical Ltd., Great Malvern, UK) was used to observe the GtDENs suspension. NTA Nanosight LM10 software 2.3 was used to measure GtDENs in the photo 11 frames per sample in triplicate, and analyzed and presented their mean and distribuion properties.

### 2.4 Phytochemical component analysis in GtDENs

Five types of components known to be contained in green tea leaves, including EC, ECF, EGC, EGCG, and Caffeine, were analyzed liquid chromatography/tandem mass spectrometry (LC-MS/MS). In details, the GtDENs solution was filtered through 0.22 µm mixed cellulose ester membrane filter (Millipore®, Burlington, MA, United States), and the filtrate was analyzed. Xevo TQ Absolute system (Waters Corp., Milford, MA, United States) with a ACQUITY UPLC BEH C_18_ column (130 Å, 1.7 μm, 2.1 mm × 100 mm; Waters Corp.) was used for separation. The column temperature was set at 40 °C, and the flow rate was 0.3 mL/min. The mobile phases were 0.1% (v/v) formic acid in water (solvent A) and acetonitrile (solvent B). A linear gradient was as follows: 20% B, 20% B, 95% B, 100% B, 20% B, and 20% B at 0.0, 0.1, 14.0, 15.0, 15.1, and 18 min, respectively. Mass detection of precursors and fragments was performed under the following conditions. Ionization mode: positive and negative; MS scan type: full scan and single ion recording; ionization source: ESI; MS scan range: 200–2,000 mass-to-charge ratio (*m/z*); MS/MS scan range: 30–2,000 *m/z*; capillary voltage: 3.3 kV; extractor voltage: 3 V; RF lens voltage: 0.3 V; source temperature: 120 °C; desolvation temperature: 300 °C; desolvation gas: 600 L/h; cone gas: 50 L/h; ion spray voltage: 5.5 kV and 4.5 kV (positive and negative, respectively); collision energy: 10 and −10 (positive and negative, respectively); collision gas (N_2_): 0.14 mL/min.

### 2.5 Cell culture

HaCaT cells and HEK293T cells were incubated in DMEM supplemented with 10% and 5% fetal bovine serum (FBS, Gibco, Grand Island, UT, United States), respectively, and 1% penicillin and streptomycin. The cells were grown in a humidified incubator with 5% CO_2_ at 37 °C.

### 2.6 Cell viability assay

HaCaT cells were seeded in a 96-well plate at a density of 2 × 10^5^ cells/mL and incubated for 18 h. Then the cells were treated with GtDENs at concentrations of 2.5 × 10^9^, 5 × 10^9^, 1 × 10^10^, 2 × 10^10^, and 4 × 10^10^ particles/mL. After 24 h, the cell viability was determined by conventional MTT assay.

### 2.7 ROS generation assay

HaCaT cells were seeded in a 6-well plate at density of 2 × 10^5^ cells/mL and incubated for 18 h. The cells were pre-treated with GtDENs for 30 min and treated with H_2_O_2_ (600 μM) or UVB (30 mJ/cm^2^). After 24 h, the cells were stained with 10 µM H2DCFDA and incubated for 20 min in the dark. Then, the cells were fixed in a 4% formaldehyde solution and stained with 4′,6-diamidino-2-phenylindole (DAPI). Photographs were captured using a Nikon Eclipse Ti (Nikon, Japan) fluorescence microscope. For flow cytometry, HaCaT cells were treated with GtDENs and H_2_O_2_ or UVB in the same manner as mentioned above. After 24 h, cells were harvested and resuspended in 300 μL of phosphate-buffered saline. And H2DCFDA was added to the cells to a concentration of 10 μM, and the cells were incubated for 20 min in the dark. The fluorescence was detected at 485/535 nm using a flow cytometer (Beckman Coulter, Brea, CA, United States).

### 2.8 SA-b gal assay

HaCaT cells were seeded in 6 cm plate at a concentration of 2 × 10^5^ cells/mL and incubated for 18 h in an incubator. The seeded cells were pre-treated with GtDENs for 30 min and then treated with t-BHP at a concentration of 50 mM. After 1 h, the cells were washed with PBS and new fresh media were treated. After 4 h, this procedure was repeated and the cells were kept in the fresh media overnight. Those two times treatments of GtDENs and t-BHP were repeated for 4 days and the β-galactosidase staining was conducted with the instructions of senescence β-galactosidase staining kit from Cell Signaling Technology (Beverly, MA, United States; Catalog No: 9860).

### 2.9 RNA extraction and quantitative real-time polymerase chain reaction (qPCR)

HaCaT cells were seeded in a 6-well plate at 2 × 10^5^ cells/mL and incubated for 18 h in an incubator. The seeded cells were pre-treated with GtDENs for 30 min and then treated with H_2_O_2_ (600 µM). After 24 h, the cells were harvested, and the total RNA was extracted with TRIzol reagent according to the manufacturer’s instructions. The complementary DNA was synthesized with a cDNA synthesis kit. Finally, qRT-PCR was performed with Pcrbio’s qPCRBIO SyGreen mix. The primers used in this study are listed in Supplementary Material (Supplementary Table S1).

### 2.10 Preparation of cell lysates and immunoblotting

HaCaT cells or HEK293T cells were lysed with lysis buffer to get the whole lysates. The lysis buffer consisted of 50 mM Tris-HCl (pH 7.5), 120 mM NaCl, 25 mM β-glycerol phosphate (pH 7.5), 20 mM NaF, 2% NP-40, 2 μg/mL leupeptin, 2 μg/mL pepstatin A, 1 mM benzamide, 2 μg/mL aprotinin, 1.6 mM pervanadate, 100 µM phenylmethylsulfonyl fluoride, and 100 µM Na_3_VO_4_. Samples containing equal amounts of proteins (20 μg) were loaded in polyacrylamide gels and separated by size with sodium dodecyl sulfate polyacrylamide gel electrophoresis, followed by transfer of proteins to polyvinylidene fluoride (PVDF) membranes. The primary antibody was diluted 1:2,500 with tris-buffered saline containing 0.1% Tween® 20 detergent (TBST) and 3% BSA, and the antibody was incubated on the PVDF membrane at 4 °C for 18 h. After washing three times with TBST, the secondary antibodies were incubated at a ratio of 1:2500 for 90 min. After washing three times with TBST, the immunoreactive bands were detected by enhanced peroxidase with an ELPIS-BIOTECH in a chemidoc of ATTO.

### 2.11 Molecular docking models

For molecular docking analysis, the X-ray crystallographic structure of human p38 MAPK (MAPK14) was obtained from the Protein Data Bank (PDB; PDB ID: 5UOJ). The three-dimensional structure of caffeine was retrieved from PubChem (CID: 2519). Molecular docking simulations were performed using AutoDock Vina, and the docking grid was defined to encompass the ATP-binding pocket of p38 MAPK. Binding poses were ranked based on predicted binding affinity scores.

### 2.12 CETSA

Cellular thermal shift assay was performed using HEK293T cells transfected with p38 as previously described (You et al., 2024). HEK293T cell lysates were prepared from GtDENs or caffeine or DW-treated cells. Thermal stability of p38 was confirmed by immunoblotting analysis with p38 antibodies. The band intensity was quantified with ImageJ.

### 2.13 Statistical analysis

All quantitative data are presented as mean ± standard deviation (SD) from at least three independent biological experiments. Normality of data distribution was assessed using the Shapiro–Wilk test. For comparisons among multiple groups, one-way analysis of variance (ANOVA) followed by Tukey’s *post hoc* test was applied. Exact p-values are reported in the figure legends. Statistical significance was defined as p < 0.05.

## 3 Results

### 3.1 Physical characteristics and phytochemical components of GtDENs

**FIGURE 1:**
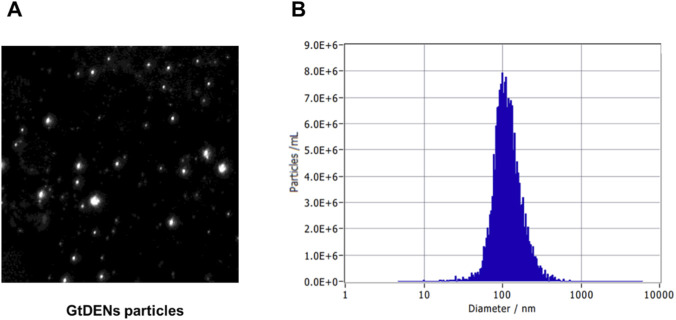
Physicochemical characterization of green tea–derived exosome-like nanoparticles (GtDENs). **(A)** Representative nanoparticle tracking analysis (NTA) image of GtDENs. **(B)** Size distribution profile of GtDENs measured by NTA, showing a predominant nanoparticle population within the expected exosome-like size range. The particle concentration and mean size were determined from independent measurements.

The external images of GtDENs were captured by ZetaVIEW, a nanoparticle tracking analyzer (NTA) (Figure 1A). The particle numbers based on the size of GtDENs were analyzed with the same instrument (Figure 1B). The mean size was 110 nm and the concentration was 1.2 × 10^11^ particles/mL. For additional analysis of GtDENs and phytochemical components inside, 3 more batches of GtDENs were independently prepared. Each particle concentration of GtDENs batch was 5.20 × 10^11^, 5.10 × 10^11^, and 4.10 × 10^11^ particles/mL, respectively. The initial GtDEN preparation showed a mean size of ∼110 nm, whereas three independently prepared batches exhibited mean sizes ranging from 139 to 143 nm, reflecting batch-to-batch variability commonly observed in plant-derived nanoparticles (Table 1). The phytochemical components in GtDENs were also measured three times independently (Table 2). Epicatechin (EC), epicatechin gallate (ECG), and Caffeine were detected inside GtDENs, but epigallocatechin (EGC) and epigallocatechin gallate (EGCG) were not detected. EC and ECG were detected very little, and the majority of the components in GtDENs was caffeine of which amount was 940.3, 1017.7, and 1025.0 μg/mL, respectively (Table 2).

**TABLE 1:** Particle concentration and mean size of independently prepared GtDEN batches.

GtDENs-ID	GtDEN 1	GtDEN 2	GtDEN 3
Particle concentration (particles/mL)	5.20 × 10^11^	5.10 × 10^11^	4.10 × 10^11^
Mean size (nm)	139.7	140.2	142.8

Green tea–derived exosome-like nanoparticles (GtDENs) were independently prepared in three batches. Particle concentration and mean size were determined by nanoparticle tracking analysis (NTA). Data represent mean values obtained from independent measurements.

**TABLE 2:** Phytochemical components encapsulated within GtDENs.

Phytochemical-ID	GtDEN 1	GtDEN 2	GtDEN 3
EC (μg/mL)	1.6	1.7	1.6
ECG (μg/mL)	0.03	0.03	0.03
EGC (μg/mL)	ND^a^	ND	ND
EGCG (μg/mL)	ND	ND	ND
Caffeine (μg/mL)	940.3	1,017.7	1,025.0

Phytochemical constituents of GtDENs were quantified by LC–MS/MS analysis. Concentrations are expressed as μg/mL of the GtDEN suspension. ND indicates not detected.

^a^ ND: not detected.

### 3.2 GtDENs protect keratinocytes from oxidative stress–induced cytotoxicity and ROS accumulation

**FIGURE 2:**
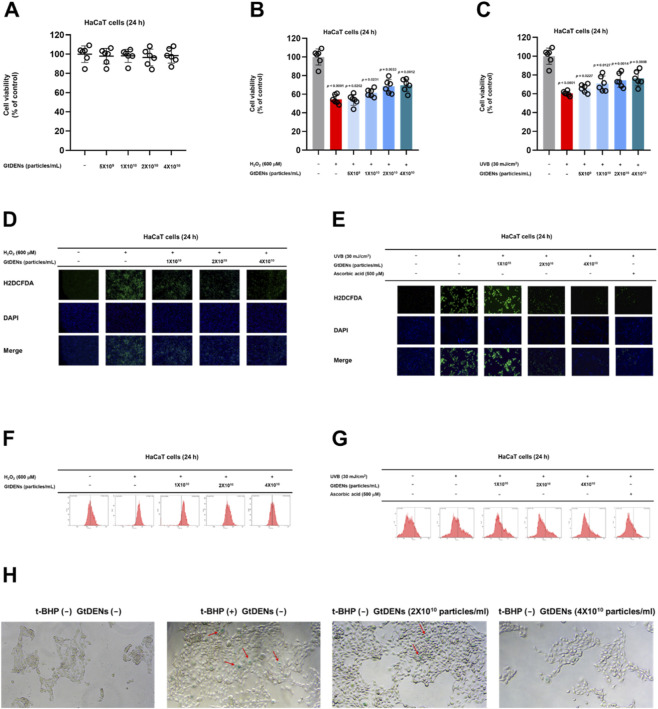
GtDENs protect keratinocytes from oxidative stress–induced cytotoxicity, ROS accumulation, and cellular senescence. **(A)** HaCaT cells were treated with increasing concentrations of GtDENs for 24 h, and cell viability was assessed by MTT assay. **(B,C)** Cells were pretreated with GtDENs and subsequently exposed to hydrogen peroxide (H_2_O_2_, 600 μM) or UVB irradiation (30 mJ/cm^2^). Cell viability was determined after 24 h. **(D,E)** Intracellular reactive oxygen species (ROS) levels following H_2_O_2_ exposure were assessed using H_2_DCFDA staining. Representative fluorescence images and flow cytometric quantification are shown. **(F,G)** ROS accumulation following UVB exposure was evaluated by H_2_DCFDA staining and quantified by flow cytometry. **(H)** Cellular senescence was induced by repeated tert-butyl hydroperoxide (t-BHP) treatment and assessed by senescence-associated β-galactosidase (SA-β-gal) staining. Data represent mean ± SD (n = 3 independent biological replicates). Statistical analysis was performed using one-way ANOVA followed by Tukey’s *post hoc* test. Exact p-values for individual comparisons are indicated in the figure panels.

To evaluate the cytoprotective effects of green tea–derived exosome-like nanoparticles (GtDENs), human keratinocyte HaCaT cells were treated with increasing concentrations of GtDENs and cell viability was assessed by MTT assay. GtDENs did not induce detectable cytotoxicity at concentrations up to 4 × 10^10^ particles/mL, indicating good cellular tolerability (Figure 2A).

Next, the protective effects of GtDENs against oxidative stress were examined. Exposure to hydrogen peroxide (H_2_O_2_, 600 μM) or ultraviolet B (UVB, 30 mJ/cm^2^) significantly reduced HaCaT cell viability. Pretreatment with GtDENs dose-dependently attenuated oxidative stress–induced cytotoxicity under both conditions (Figures 2B,C), demonstrating a protective effect against chemically and physically induced oxidative insults.

To determine whether this protection was associated with modulation of intracellular ROS levels, ROS generation was quantified using H_2_DCFDA staining. H_2_O_2_ or UVB exposure markedly increased intracellular ROS accumulation, as visualized by fluorescence microscopy and quantified by flow cytometry (Figures 2D–G). Pretreatment with GtDENs significantly reduced ROS levels in a dose-dependent manner under both oxidative stress conditions. Ascorbic acid was used as a positive control and showed comparable ROS-scavenging activity.

These results indicate that GtDENs protect keratinocytes from oxidative stress–induced cytotoxicity, at least in part, through suppression of intracellular ROS accumulation.

### 3.3 GtDENs suppress oxidative stress–induced cellular senescence and apoptosis

Because sustained oxidative stress is a major driver of cellular senescence and apoptosis, we next examined whether GtDENs modulate these downstream cellular outcomes. Senescence-associated β-galactosidase (SA-β-gal) staining was performed following repeated tert-butyl hydroperoxide (t-BHP) treatment to induce stress-related senescence. t-BHP exposure markedly increased SA-β-gal–positive cells, whereas GtDENs treatment reduced senescence-associated staining in a dose-dependent manner (Figure 2H).

**FIGURE 3:**
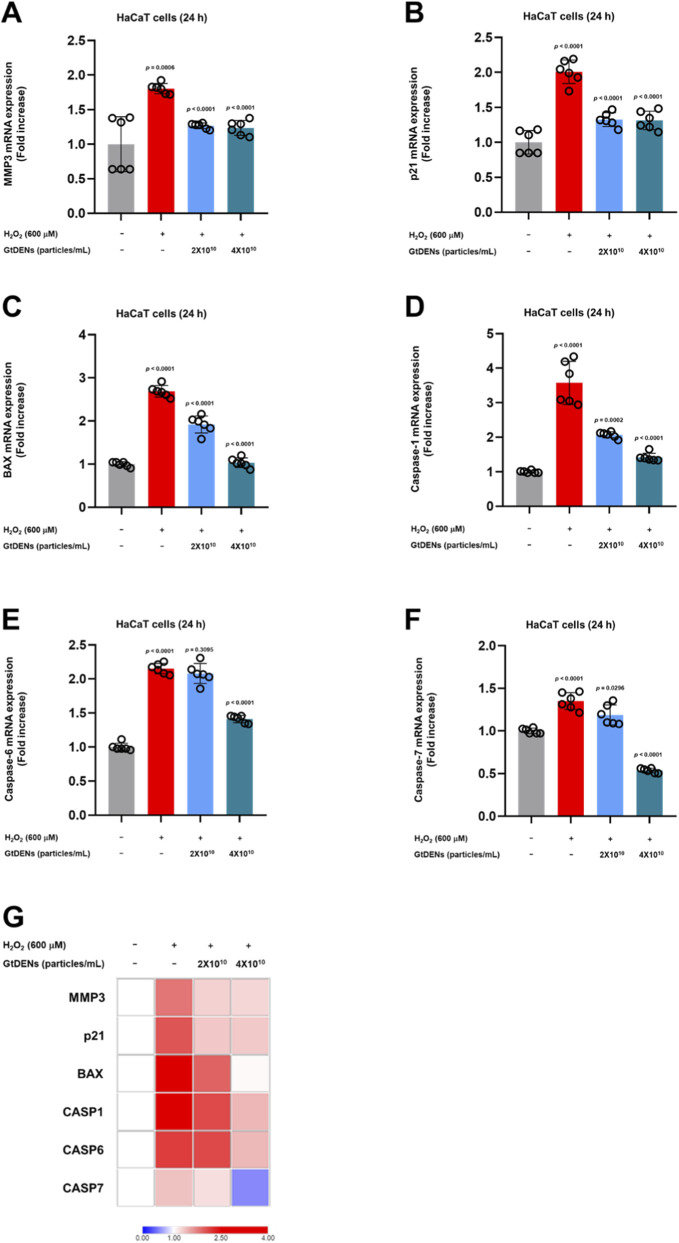
GtDENs suppress oxidative stress–induced expression of senescence- and apoptosis-related genes. **(A,B)** mRNA expression levels of MMP3 and p21 following H_2_O_2_ exposure were quantified by real-time PCR. **(C–F)** Expression levels of apoptosis-related genes (BAX, CASP1, CASP6, CASP7) were analyzed. **(G)** Heatmap summarizing relative changes in gene expression across experimental groups. Data represent mean ± SD (n = 3 independent biological replicates). Statistical analysis was performed using one-way ANOVA followed by Tukey’s *post hoc* test. Exact p-values for individual comparisons are indicated in the figure panels.

To further characterize the effects of GtDENs at the transcriptional level, the expression of senescence- and apoptosis-related genes was analyzed by quantitative real-time PCR following H_2_O_2_ exposure. Oxidative stress significantly upregulated the expression of matrix metalloproteinase 3 (MMP3) and the cyclin-dependent kinase inhibitor p21, both of which are associated with cellular aging and growth arrest. Pretreatment with GtDENs significantly attenuated the induction of these genes (Figures 3A,B).

In parallel, oxidative stress increased the expression of apoptosis-related genes, including BAX and caspases (CASP1, CASP6, and CASP7). GtDENs treatment markedly suppressed the upregulation of these pro-apoptotic transcripts induced by H_2_O_2_ (Figures 3C–F). A heatmap summarizing the overall gene expression changes further illustrates the coordinated suppression of senescence- and apoptosis-associated genes by GtDENs under oxidative stress conditions (Figure 3G).

Collectively, these results demonstrate that GtDENs mitigate oxidative stress–induced cellular senescence and apoptotic signaling in human keratinocytes.

### 3.4 GtDENs preferentially suppress p38 MAPK–associated AP-1 signaling under oxidative stress

**FIGURE 4:**
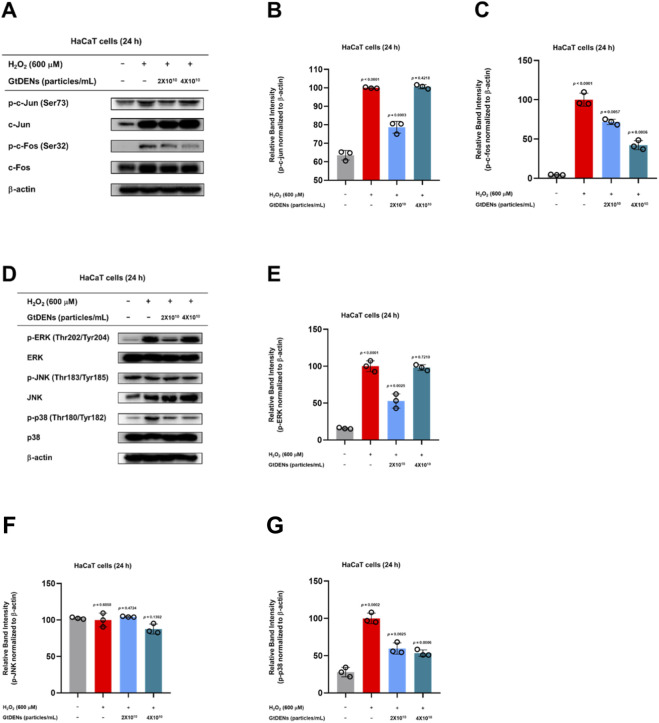
GtDENs preferentially suppress p38 MAPK–associated AP-1 signaling under oxidative stress. **(A–C)** Phosphorylation and total protein levels of c-Jun and c-Fos were analyzed by immunoblotting. **(D–G)** Phosphorylation and total protein levels of ERK, JNK, and p38 MAPK were assessed. Representative immunoblots and densitometric quantification are shown. Phosphorylated protein levels were normalized to total protein and β-actin. Data represent mean ± SD (n = 3 independent biological replicates). Statistical analysis was performed using one-way ANOVA followed by Tukey’s *post hoc* test. Exact p-values for individual comparisons are indicated in the figure panels.

To investigate the signaling mechanisms underlying the protective effects of GtDENs, we examined the activation status of the activator protein-1 (AP-1) pathway following oxidative stress. Exposure of HaCaT cells to H_2_O_2_ markedly increased the phosphorylation of the AP-1 subunits c-Jun and c-Fos. Pretreatment with GtDENs significantly reduced the phosphorylation of c-Fos in a dose-dependent manner, whereas no appreciable inhibitory effect was observed on c-Jun phosphorylation (Figures 4A–C).

Because AP-1 activity is regulated by upstream mitogen-activated protein kinases (MAPKs), we next assessed the phosphorylation status of ERK, JNK, and p38 MAPK. H_2_O_2_ exposure increased ERK phosphorylation; however, GtDENs did not affect ERK activation (Figures 4D,E). JNK phosphorylation remained unchanged by either H_2_O_2_ or GtDENs treatment (Figures 4D,F). In contrast, H_2_O_2_-induced phosphorylation of p38 was significantly attenuated by GtDENs in a dose-dependent manner (Figures 4D,G).

These results indicate that GtDENs preferentially suppress oxidative stress–induced activation of the p38 MAPK–AP-1 signaling axis, with preferential inhibition of p38 and downstream c-Fos phosphorylation, while sparing ERK and JNK signaling pathways.

### 3.5 Involvement of p38 MAPK signaling in mediating GtDEN activity

**FIGURE 5:**
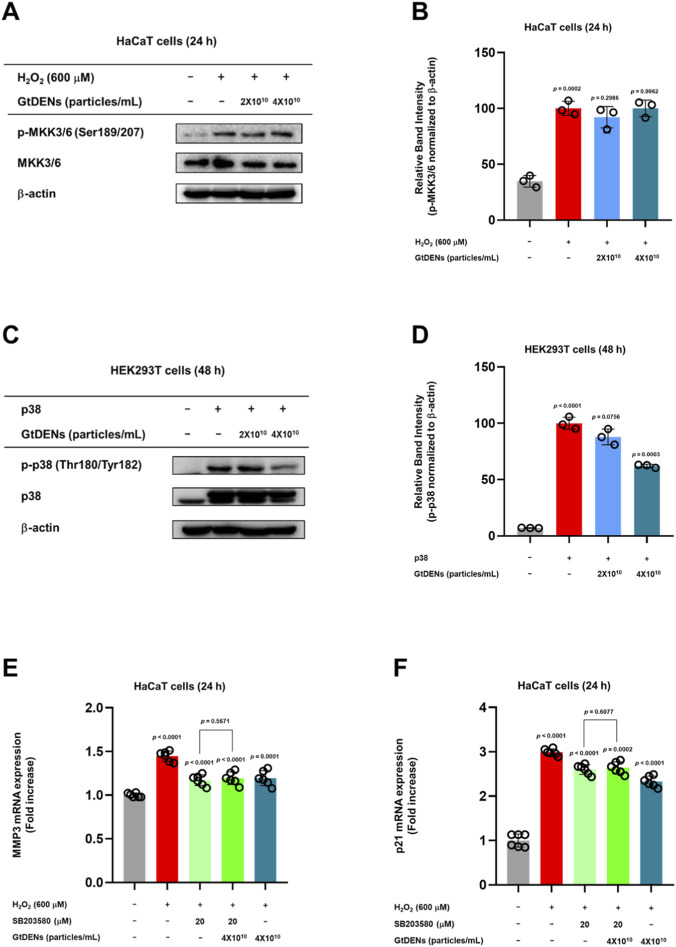
Involvement of p38 MAPK signaling in mediating GtDEN activity. **(A,B)** Phosphorylation and total protein levels of MKK3/6 were analyzed. **(C,D)** HEK293T cells transfected with p38 expression plasmid were treated with GtDENs, and phosphorylation levels were assessed. **(E,F)** HaCaT cells were treated with SB203580 or GtDENs prior to H_2_O_2_ exposure. Expression levels of p21 and MMP3 were quantified. Data represent mean ± SD (n = 3 independent biological replicates). Statistical analysis was performed using one-way ANOVA followed by Tukey’s *post hoc* test. Exact p-values for individual comparisons are indicated in the figure panels.

To determine whether p38 MAPK is directly targeted by GtDENs, we first examined the activation status of its upstream kinases, MKK3 and MKK6. H_2_O_2_ exposure significantly increased MKK3/6 phosphorylation; however, GtDENs treatment did not alter the phosphorylation levels of these upstream kinases (Figures 5A,B), suggesting that GtDENs act downstream or independently of MKK3/6.

To further validate p38 as the functional target of GtDENs, p38 was ectopically overexpressed in HEK293T cells. Overexpression of p38 resulted in increased p38 phosphorylation, which was significantly reduced by GtDENs treatment (Figures 5C,D), supporting a direct inhibitory effect of GtDENs on p38 activation.

Pharmacological inhibition experiments were performed using the selective p38 inhibitor SB203580. H_2_O_2_-induced upregulation of senescence-associated genes, including *MMP3* and *p21*, was significantly suppressed by SB203580 treatment. Importantly, co-treatment with GtDENs did not further reduce gene expression beyond the level achieved by SB203580 alone (Figures 5E,F), indicating epistasis between GtDENs and p38 inhibition.

Collectively, these results support a role for p38 MAPK signaling as a key regulatory node involved in the cytoprotective and anti-senescence effects of GtDENs under oxidative stress conditions. While the data indicate functional involvement of p38 MAPK, direct biochemical confirmation of specific target engagement was not established in the present study.

### 3.6 Caffeine partially recapitulates p38 MAPK–associated signaling modulation observed with GtDENs

Phytochemical profiling revealed caffeine as the predominant small-molecule component encapsulated within GtDENs (Table 2). To evaluate whether caffeine contributes to the observed signaling effects of GtDENs, we first estimated the equivalent caffeine concentrations delivered by GtDENs based on particle number and measured caffeine content. Accordingly, caffeine concentrations corresponding to 2.0 × 10^10^ and 4.0 × 10^10^ particles/mL of GtDENs were calculated and used for subsequent functional assays.

**FIGURE 6:**
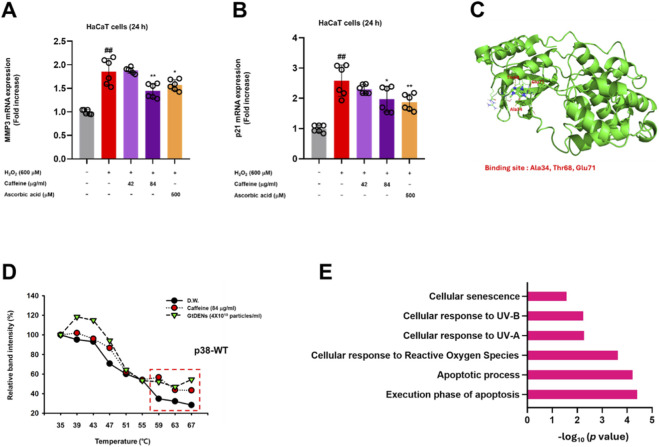
Caffeine partially recapitulates p38 MAPK–associated signaling modulation observed with GtDENs. **(A,B)** HaCaT cells were treated with caffeine at concentrations corresponding to estimated GtDEN caffeine content and exposed to H_2_O_2_. mRNA levels of MMP3 and p21 were quantified. **(C)** Molecular docking model of caffeine–p38 interaction. **(D)** CETSA analysis of p38 stability following caffeine or GtDEN treatment. **(E)** Gene ontology enrichment analysis of transcripts modulated by GtDENs. Data represent mean ± SD (n = 3 independent biological replicates). Statistical analysis for gene expression data was performed using one-way ANOVA followed by Tukey’s *post hoc* test. Exact p-values for individual comparisons are indicated in the figure panels.

Under oxidative stress conditions induced by H_2_O_2_, treatment with caffeine significantly attenuated the upregulation of senescence-associated genes MMP3 and p21 in HaCaT cells, with a more pronounced effect observed at the higher concentration (Figures 6A,B). Ascorbic acid was included as a positive control and showed comparable suppressive effects on oxidative stress–induced gene expression.

To investigate the potential interaction between caffeine and p38 MAPK, molecular docking analysis was performed. In silico docking predicted that caffeine binds within the ATP-binding pocket of p38 with a mean binding affinity of −4.8 kcal/mol, involving putative interactions with residues including Ala34, Thr68, and Glu71 (Figure 6C; Table 3).

**TABLE 3:** *In silico* binding affinity between p38 MAPK and caffeine.

Interaction	Binding affinity (kcal/mol)			
	Max	Min	Mean	Standard deviation
p38-caffeine	−5.7	−3.8	−4.8	0.57

Binding affinities between caffeine and p38 MAPK were predicted by molecular docking analysis using AutoDock Vina based on the crystal structure of human p38 MAPK obtained from the Protein Data Bank.

To further assess target engagement in a cellular context, a cellular thermal shift assay (CETSA) was conducted. Treatment with caffeine or GtDENs increased the thermal stability of wild-type p38 in HEK293T cells, as determined by immunoblotting analysis (Figure 6D), suggesting potential target engagement, although indirect stabilization effects cannot be excluded.

Finally, gene ontology analysis of transcripts modulated by GtDENs revealed enrichment of pathways related to oxidative stress response, cellular senescence, apoptosis, and MAPK signaling (Figure 6E), consistent with a p38-centered mechanism of action.

Taken together, these results indicate that caffeine contributes to the p38 MAPK–associated signaling modulation observed with GtDENs under oxidative stress conditions.

### 3.7 Exploratory analysis of small RNAs associated with GtDENs

**FIGURE 7:**
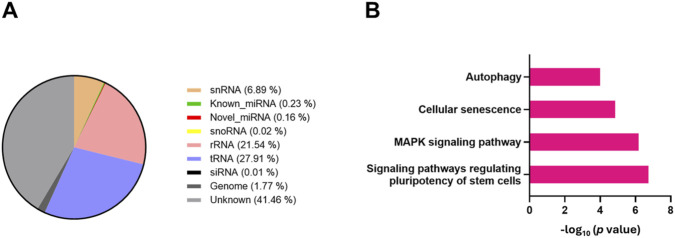
Exploratory profiling of small RNAs associated with GtDENs. **(A)** Distribution of RNA species detected by small RNA sequencing. **(B)** Gene ontology enrichment analysis of predicted targets of putative Camellia sinensis–derived miRNAs (CsmiR1–CsmiR15).

To further explore additional bioactive components within GtDENs beyond small-molecule metabolites, small RNA sequencing was performed on total RNA isolated from GtDENs. Sequence analysis revealed a diverse population of small RNAs, including small nuclear RNA (snRNA), microRNA (miRNA), small nucleolar RNA (snoRNA), ribosomal RNA (rRNA), transfer RNA (tRNA), and small interfering RNA (siRNA) (Figure 7A).

Among the detected miRNAs, several sequences corresponded to previously annotated plant miRNAs, whereas a subset represented putative novel miRNA species. These newly identified miRNAs were designated *Camellia sinensis*–derived miRNAs (CsmiR1–CsmiR15). Bioinformatic target prediction was performed for these novel miRNAs, followed by gene ontology analysis of the predicted target genes.

Gene ontology enrichment analysis indicated that the predicted targets of CsmiR1–CsmiR15 were associated with pathways related to cellular senescence, oxidative stress responses, and MAPK signaling (Figure 7B). These findings suggest that, in addition to small-molecule components such as caffeine, GtDENs may carry regulatory RNA species with the potential to modulate stress-responsive signaling pathways.

Given the exploratory nature of these analyses, the functional relevance of GtDEN-associated small RNAs in mammalian cells remains to be determined. Further studies will be required to validate their biological activity and contribution to the observed cellular effects.

## 4 Discussion

In this study, we demonstrate that green tea–derived exosome-like nanoparticles (GtDENs) exert cytoprotective and anti-senescence effects in human keratinocytes exposed to oxidative stress. GtDENs effectively reduced intracellular reactive oxygen species accumulation and attenuated oxidative stress–induced cytotoxicity, cellular senescence, and apoptotic gene expression (Nishi et al., 2017; Juan et al., 2021; Li et al., 2024). Mechanistically, these effects were associated with selective suppression of the p38 MAPK–AP-1 signaling axis, characterized by inhibition of p38 phosphorylation and downstream c-Fos activation without affecting ERK or JNK pathways (Wu et al., 2005; Zenz et al., 2008; Slomiany and Slomiany, 2013). Target validation experiments further identified p38 MAPK as a functional signaling node mediating GtDEN activity, as supported by p38 overexpression and pharmacological epistasis analyses (Iwasa et al., 2003; Zhou et al., 2010; Slomiany and Slomiany, 2013; Harada et al., 2014). In addition, caffeine, the predominant small-molecule component encapsulated within GtDENs, was suggested to engage p38 MAPK and contribute to the observed signaling inhibition (Conney et al., 2013; Takahashi and Ishigami, 2017; Ősz et al., 2022). Together, these findings provide evidence supporting the involvement of p38 MAPK signaling in mediating the effects of GtDENs under oxidative stress conditions.

Plant-derived exosome-like nanoparticles have recently gained attention as naturally occurring nanostructures capable of transferring bioactive cargos across species barriers (Regente et al., 2012; Cai et al., 2019). Unlike conventional phytochemical extracts, PDENs encapsulate complex molecular cargos within lipid bilayer membranes, enabling enhanced stability and targeted delivery to recipient cells (Mu et al., 2014; Liang et al., 2021; Malekian et al., 2023). In this context, our findings suggest that GtDENs function not merely as passive carriers of antioxidant compounds but as biologically active nanocarriers that modulate intracellular signaling pathways relevant to oxidative stress responses. The ability of GtDENs to selectively suppress p38 MAPK activation highlights a pharmacological mode of action that extends beyond non-specific radical scavenging (Slomiany and Slomiany, 2013; Song et al., 2022). Such signaling-level modulation distinguishes PDENs from soluble plant metabolites and supports their emerging role as functional nanotherapeutic platforms capable of engaging defined molecular targets within mammalian cells (Mu et al., 2014; De Robertis et al., 2020; Liang et al., 2021). While the present study did not directly visualize or quantify cellular uptake of GtDENs, the observed modulation of intracellular signaling pathways under defined treatment conditions is consistent with previous reports describing uptake of plant-derived extracellular vesicles by mammalian cells (Mu et al., 2014; Liang et al., 2021). Nevertheless, direct confirmation of GtDEN internalization and intracellular trafficking will be required in future studies to definitively establish vesicle-mediated target engagement.

It is important to acknowledge that isolation of plant-derived exosome-like nanoparticles remains technically challenging. Although an aqueous two-phase extraction method was employed and previously validated by cryo-TEM imaging, additional purification approaches such as size-exclusion chromatography or density gradient ultracentrifugation were not performed in the present study. Therefore, the potential contribution of co-isolated soluble phytochemicals cannot be fully excluded. It is recognized that comprehensive characterization of plant-derived extracellular vesicles, including ultrastructural validation and marker profiling, remains an evolving field with ongoing methodological standardization efforts. The present study therefore focuses on functional signaling outcomes while acknowledging the need for expanded structural validation in future investigations.

The selective inhibition of p38 MAPK observed in this study appears to be mechanistically and pharmacologically significant. Among MAPK family members, p38 functions as a central integrator of oxidative and environmental stress signals and plays a pivotal role in driving stress-induced cellular senescence, growth arrest, and inflammatory gene expression (Iwasa et al., 2003; Zhou et al., 2010; Slomiany and Slomiany, 2013; Harada et al., 2014). In contrast, ERK and JNK signaling pathways are more broadly involved in mitogenic signaling and acute stress responses (Zenz et al., 2008; Harada et al., 2014), and their indiscriminate inhibition may lead to undesirable effects on cell survival and homeostasis (Lu and Xu, 2006; Tafesh-Edwards and Eleftherianos, 2020). The finding that GtDENs suppress p38 activation without affecting ERK or JNK suggests a degree of signaling specificity that is rarely achieved by conventional antioxidant compounds (Song et al., 2022). This selective modulation of p38–AP-1 signaling provides a plausible explanation for the observed attenuation of senescence- and apoptosis-associated gene expression while preserving basal cellular viability (Jacobs-Helber et al., 1998; Iwasa et al., 2003). From a pharmacological perspective, such pathway selectivity is particularly relevant for interventions aimed at mitigating chronic oxidative stress, where partial and targeted suppression of maladaptive signaling is preferable to global inhibition of stress responses (Martindale and Holbrook, 2002; Canovas and Nebreda, 2021).

Caffeine emerged as a key small-molecule mediator contributing to the p38 MAPK–associated signaling modulation observed with GtDENs. Our data suggest that caffeine may interact with p38 MAPK, as supported by molecular docking analysis and cellular thermal shift assay results demonstrating increased thermal stability of wild-type p38 in the presence of caffeine or GtDENs. These findings are consistent with previous reports describing the antioxidant and photoprotective properties of caffeine in skin and other tissues (Conney et al., 2013; Cappelletti et al., 2015; Takahashi and Ishigami, 2017; Ősz et al., 2022). While the docking predictions and CETSA results support the possibility of direct target engagement, additional structural or biochemical validation would be required to definitively establish binding specificity and interaction dynamics. Importantly, the term “selective modulation” in the present study refers to the relative attenuation of p38 phosphorylation compared to ERK and JNK under the specific experimental conditions tested, rather than exclusive biochemical specificity at the molecular level.

Although caffeine was quantified within the nanoparticle preparation, we did not perform selective depletion or dialysis experiments to completely remove free caffeine from the preparation. Accordingly, the current data should be interpreted as demonstrating functional involvement of caffeine within the GtDEN preparation rather than establishing vesicle-specific encapsulation-dependent activity. Consequently, some of the observed biological effects may reflect combined contributions of nanoparticle-associated and soluble caffeine. Future studies will be necessary to distinguish vesicle-encapsulated versus freely diffusible small-molecule contributions.

Importantly, the biological effects of GtDENs are unlikely to be solely attributable to caffeine. GtDENs represent a nanoparticle-based delivery system encapsulating multiple bioactive components, which may collectively influence intracellular signaling pathways. The lipid bilayer structure and cargo composition of plant-derived exosome-like nanoparticles may affect cellular uptake, intracellular distribution, and signaling modulation (Liang et al., 2021; Malekian et al., 2023). Therefore, while caffeine appears to contribute to p38-associated signaling regulation, the integrated nanoparticle architecture of GtDENs likely plays a critical role in shaping the magnitude and specificity of the observed effects.

In addition to small-molecule metabolites, GtDENs were found to contain diverse classes of small RNAs, including a subset of putative novel *Camellia sinensis*–derived miRNAs (Regente et al., 2012; Cai et al., 2019). Bioinformatic analyses suggested that the predicted targets of these miRNAs are enriched in pathways related to oxidative stress responses, cellular senescence, and MAPK signaling (Yu et al., 2023). While these observations raise the possibility that RNA cargos within GtDENs may contribute to stress-responsive signaling modulation, the present study was not designed to establish a direct functional role for these RNAs in mammalian cells. Accordingly, the small RNA findings should be interpreted as exploratory and hypothesis-generating (Witwer and Halushka, 2016). Future studies involving targeted validation of miRNA uptake, stability, and gene regulatory activity will be necessary to clarify their contribution to the biological effects of GtDENs (Decker, 1998). Nonetheless, the coexistence of small-molecule and RNA cargos within a single plant-derived nanoparticle highlights the potential of PDENs as multifunctional bioactive platforms capable of coordinating complex cellular responses (Mu et al., 2014; De Robertis et al., 2020; Liang et al., 2021; Malekian et al., 2023).

Although the present study primarily evaluated transcriptional regulation of senescence- and apoptosis-associated genes, future investigations incorporating protein-level quantification of key downstream targets such as MMP3 and p21 would further strengthen the mechanistic framework.

Importantly, the current findings define a functional signaling framework rather than a definitive molecular binding model. The study was designed to identify stress-responsive pathway modulation under controlled cellular conditions, and therefore prioritizes signaling-level resolution over exhaustive structural validation. Within this defined experimental scope, the data consistently support p38 MAPK–associated signaling as a central axis modulated by GtDENs.

Together, our findings provide evidence that green tea–derived exosome-like nanoparticles modulate oxidative stress–responsive signaling in human keratinocytes. The data support involvement of p38 MAPK signaling as a central axis in this response. However, further biochemical and structural studies will be required to definitively establish direct molecular interactions and to clarify the relative contributions of nanoparticle architecture and individual phytochemical components.

## Data Availability

The datasets presented in this study can be found in online repositories. The names of the repository/repositories and accession number(s) can be found in the article/Supplementary Material.
