# Supplementary material for: Green tea–derived exosome-like nanoparticles attenuate oxidative stress–induced skin senescence via modulation of p38 MAPK signaling
Source: Front Pharmacol. 2026 Apr 28;17:1806328. doi: 10.3389/fphar.2026.1806328 (PMC13160913; doi:10.3389/fphar.2026.1806328)
Supplement: Supplementary file 1 [file Table1.docx]

**TABLE S1 Primer sequences used for quantitative real-time PCR.**

| **Gene** | **Species** | **Direction** | **Sequence** |
| --- | --- | --- | --- |
| BAX | Human | F | TGATGGACGGGTCCGGG |
|  |  | R | TGTCCAGCCCATGATGGTTC |
| CASP1 | Human | F | GAAAAGCCATGGCCGACAAG |
|  |  | R | GCTGTCAGAGGTCTTGTGCT |
| CASP6 | Human | F | GAAGAGGAGGGCAAGGTGTC |
|  |  | R | CACACACAAAGCAATCGGCA |
| CASP7 | Human | F | GAGCAGGGGGTTGAGGATTC |
|  |  | R | GTCTTTTCCGTGCTCCTCCA |
| MMP3 | Human | F | TGAGGACACCAGCATGAACC |
|  |  | R | ACTTCGGGATGCCAGGAAAG |
| P21 | Human | F | AGTCAGTTCCTTGTGGAGCC |
|  |  | R | AGGAGAACACGGGATGAGGA |

All primers were designed to amplify human gene transcripts and were validated for specificity by melt-curve analysis.
