# Supplementary material for: Green tea–derived exosome-like nanoparticles attenuate oxidative stress–induced skin senescence via modulation of p38 MAPK signaling
Source: Front Pharmacol. 2026 Apr 28;17:1806328. doi: 10.3389/fphar.2026.1806328 (PMC13160913; doi:10.3389/fphar.2026.1806328)
Supplement: Supplementary file 2 [file Presentation1.pptx]

## Slide 1
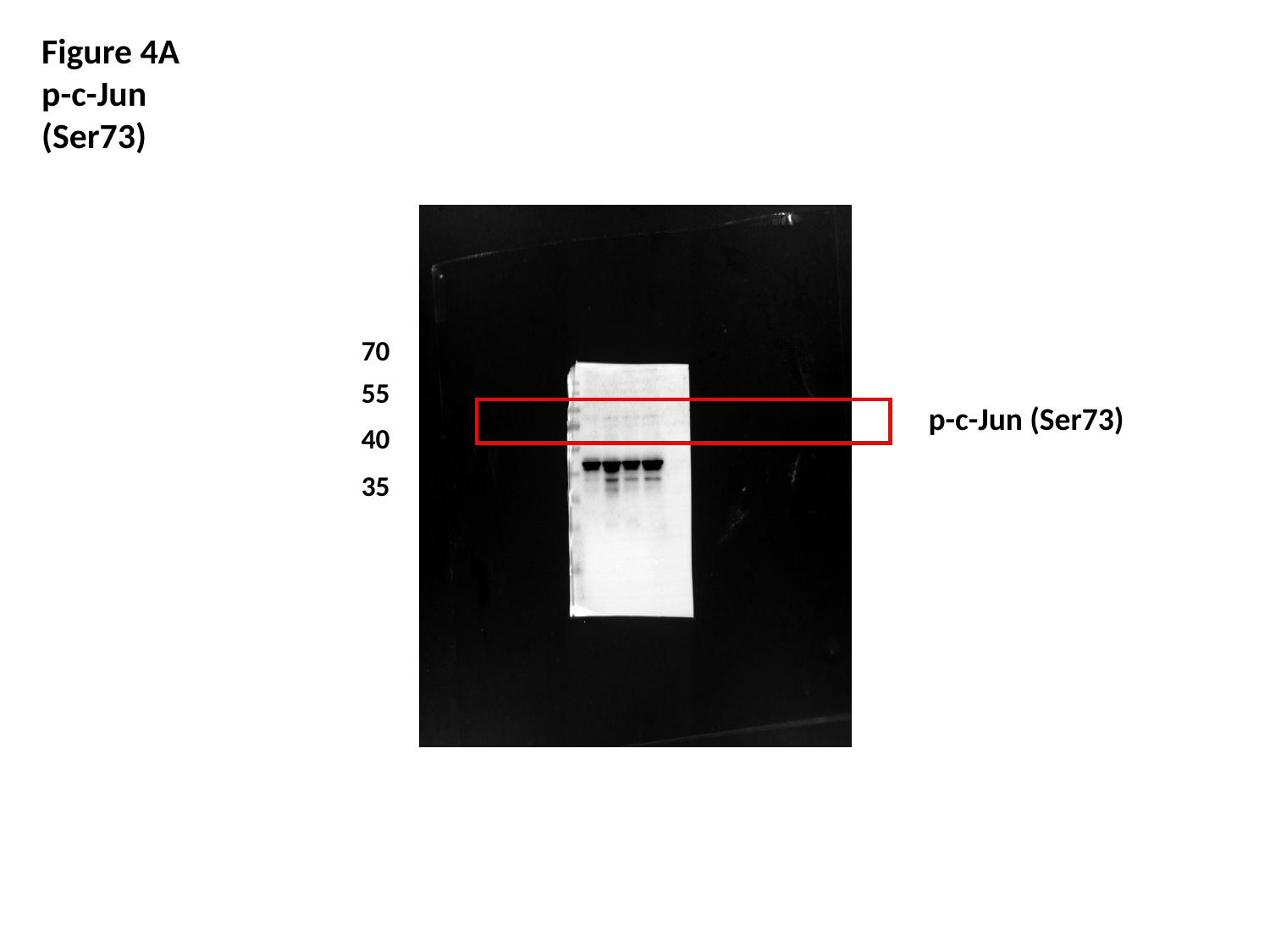

Figure 4A
p-c-Jun (Ser73)
70
55
p-c-Jun (Ser73)
40
35

## Slide 2
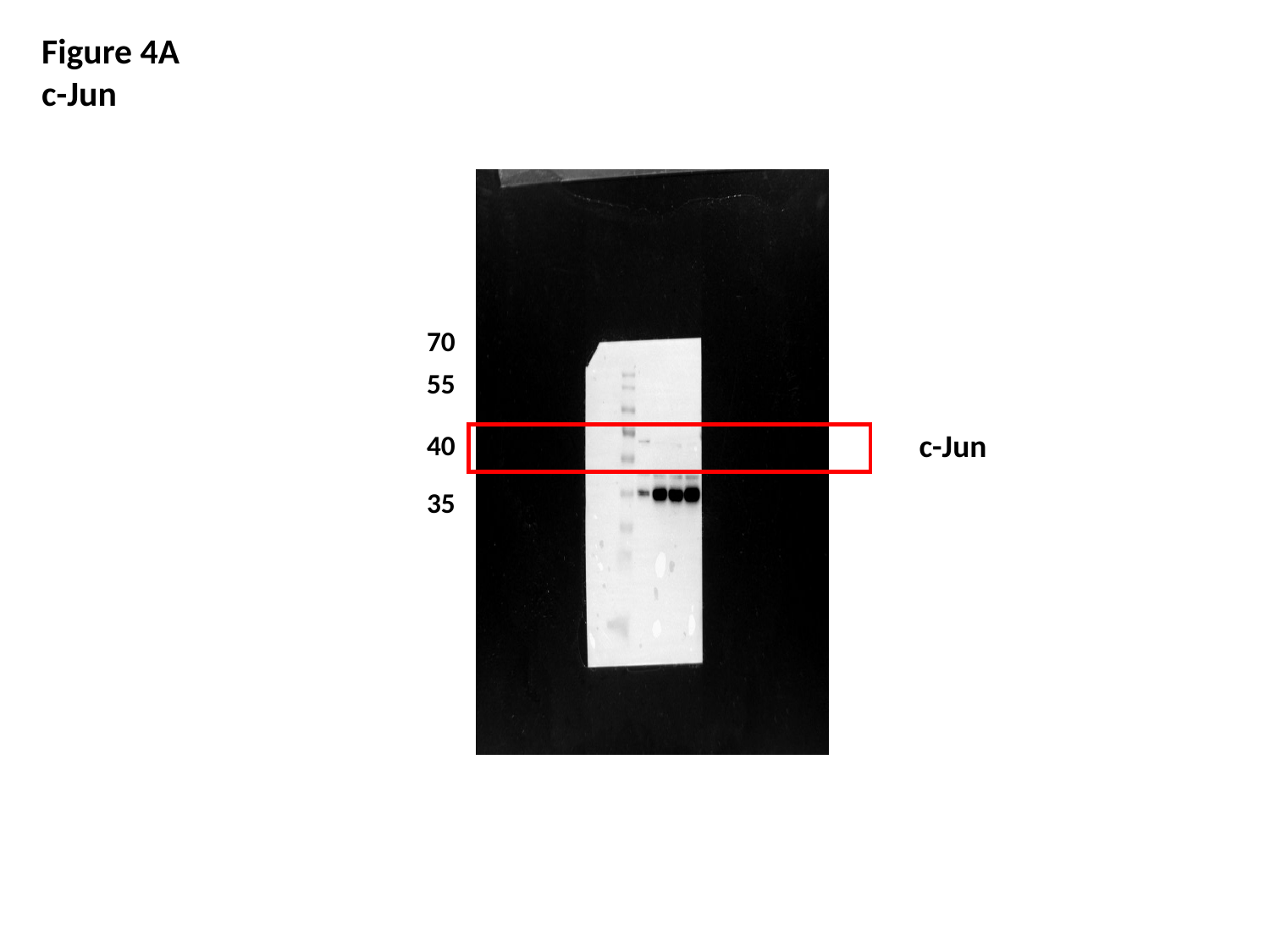

Figure 4A
c-Jun
70
55
c-Jun
40
35

## Slide 3
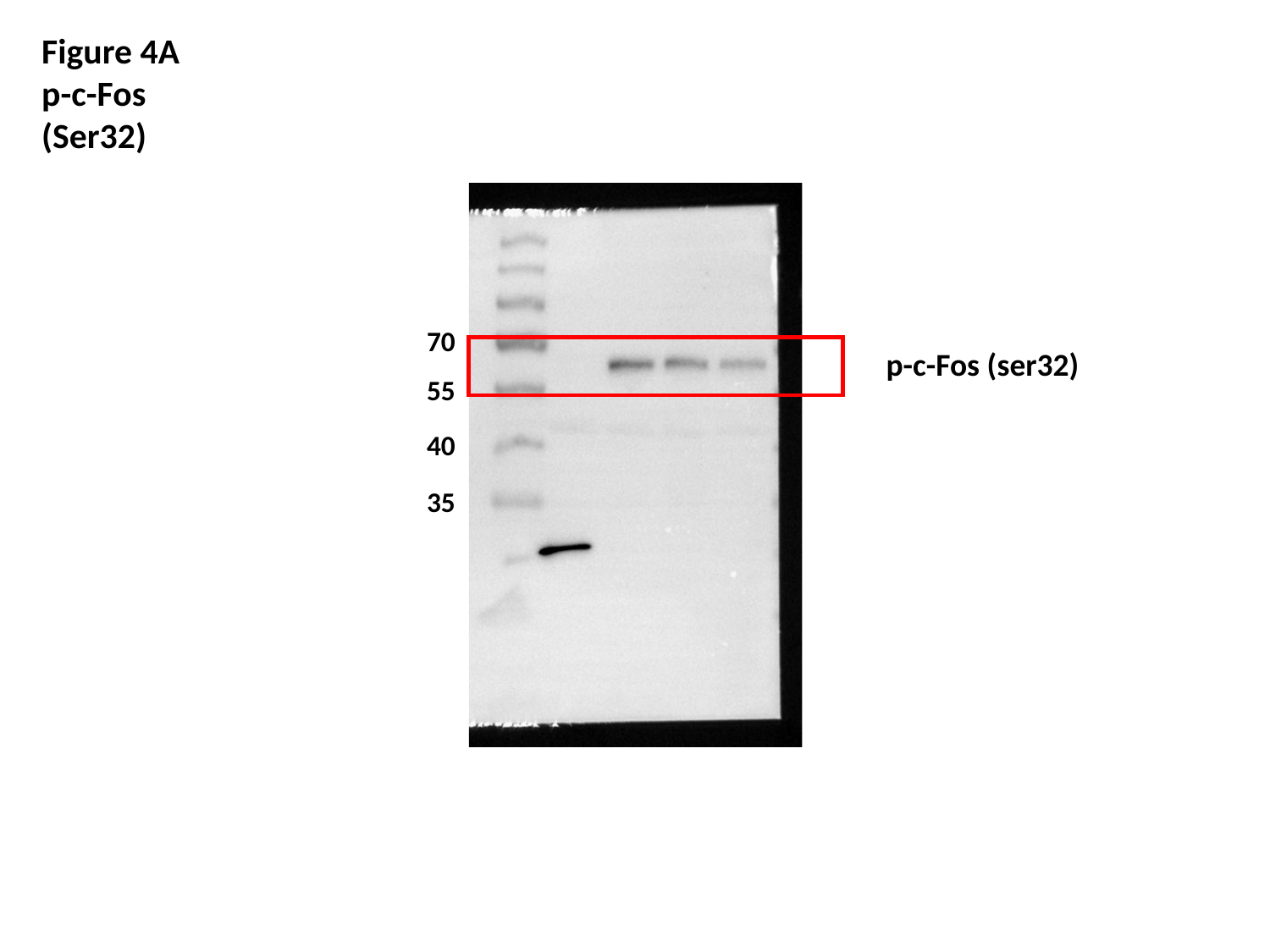

Figure 4A
p-c-Fos (Ser32)
70
p-c-Fos (ser32)
55
40
35

## Slide 4
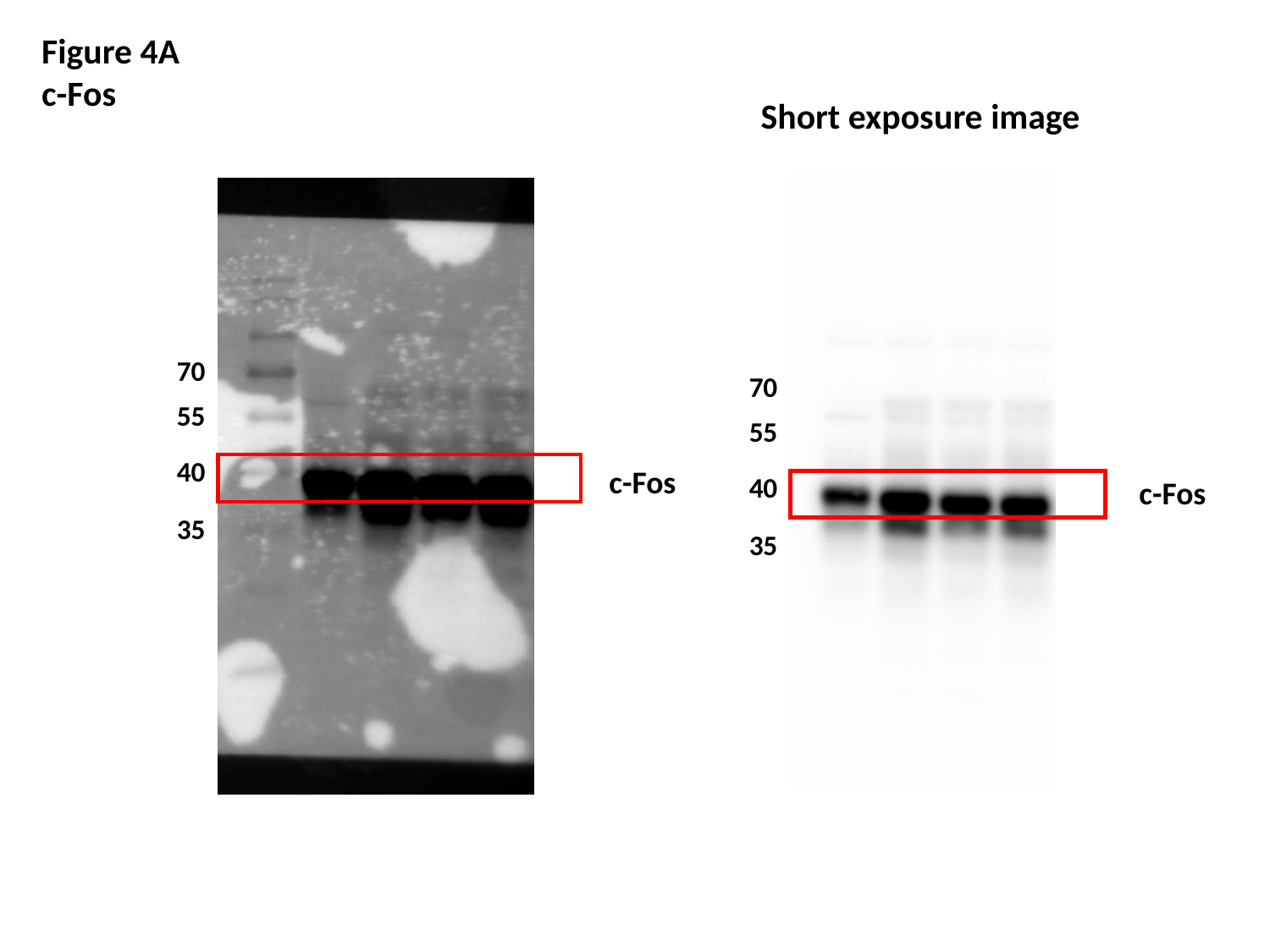

Figure 4A
c-Fos
Short exposure image
70
70
55
55
40
c-Fos
40
c-Fos
35
35

## Slide 5
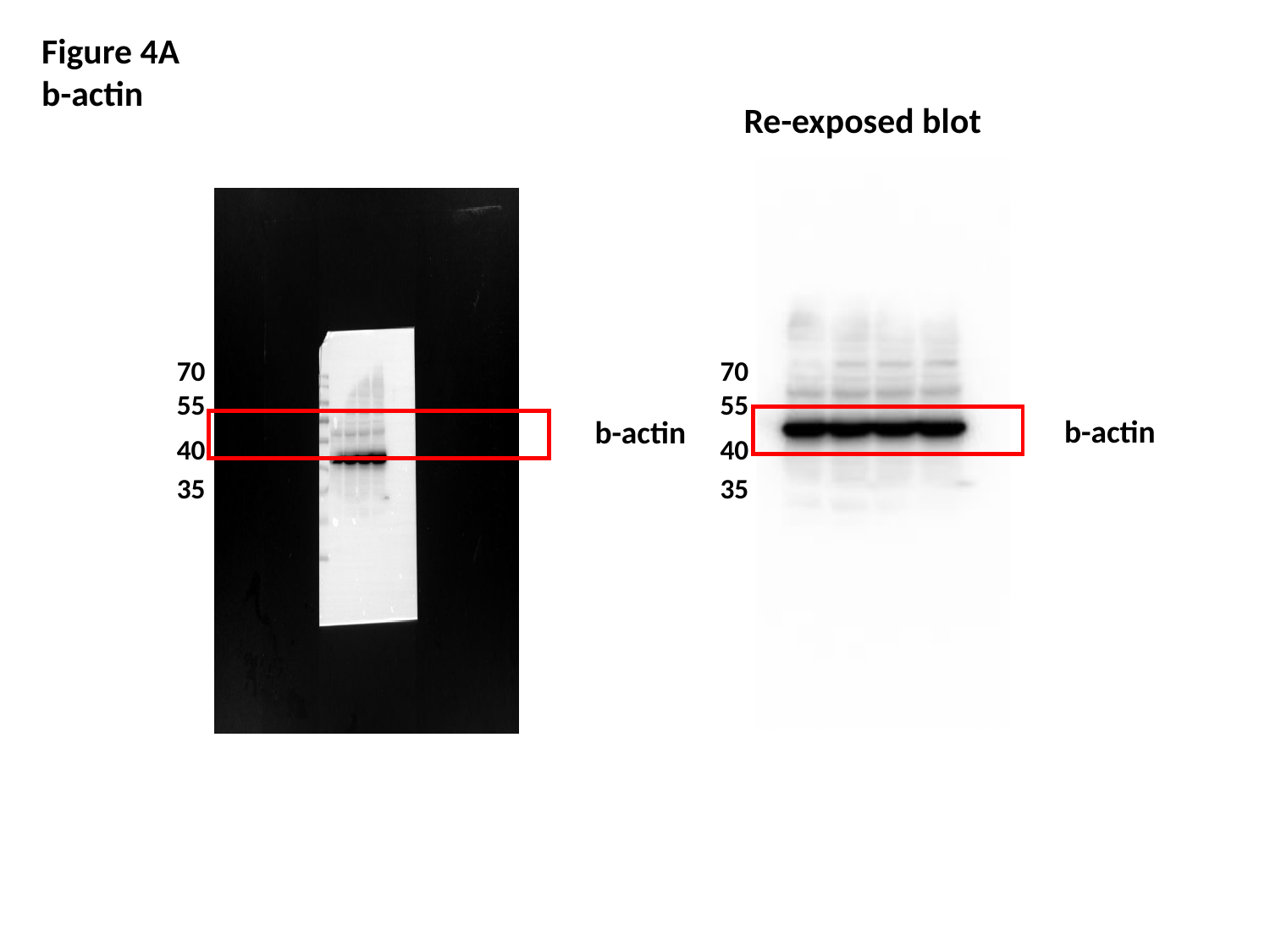

Figure 4A
b-actin
Re-exposed blot
70
70
55
55
b-actin
b-actin
40
40
35
35

## Slide 6
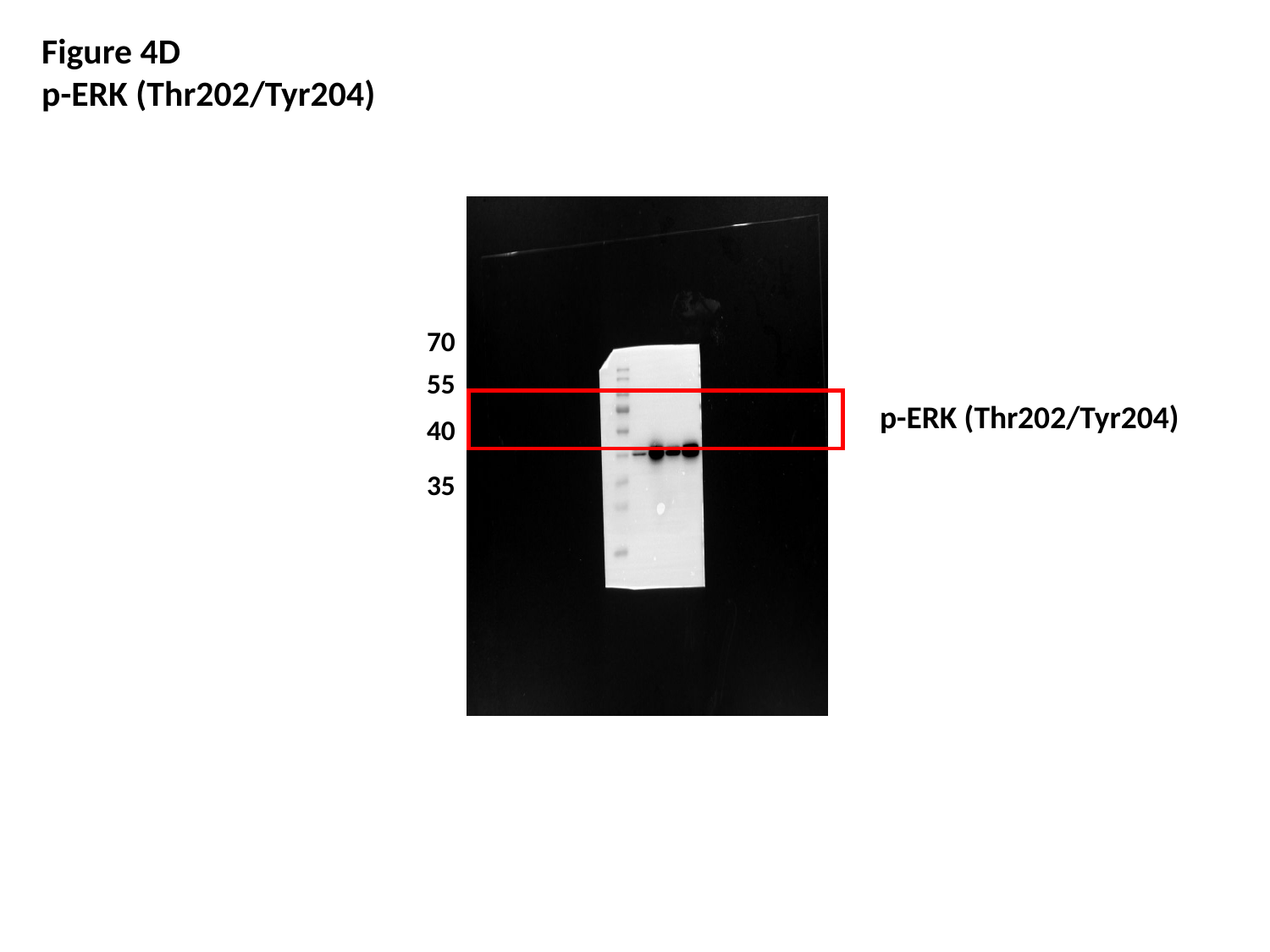

Figure 4D
p-ERK (Thr202/Tyr204)
70
55
p-ERK (Thr202/Tyr204)
40
35

## Slide 7
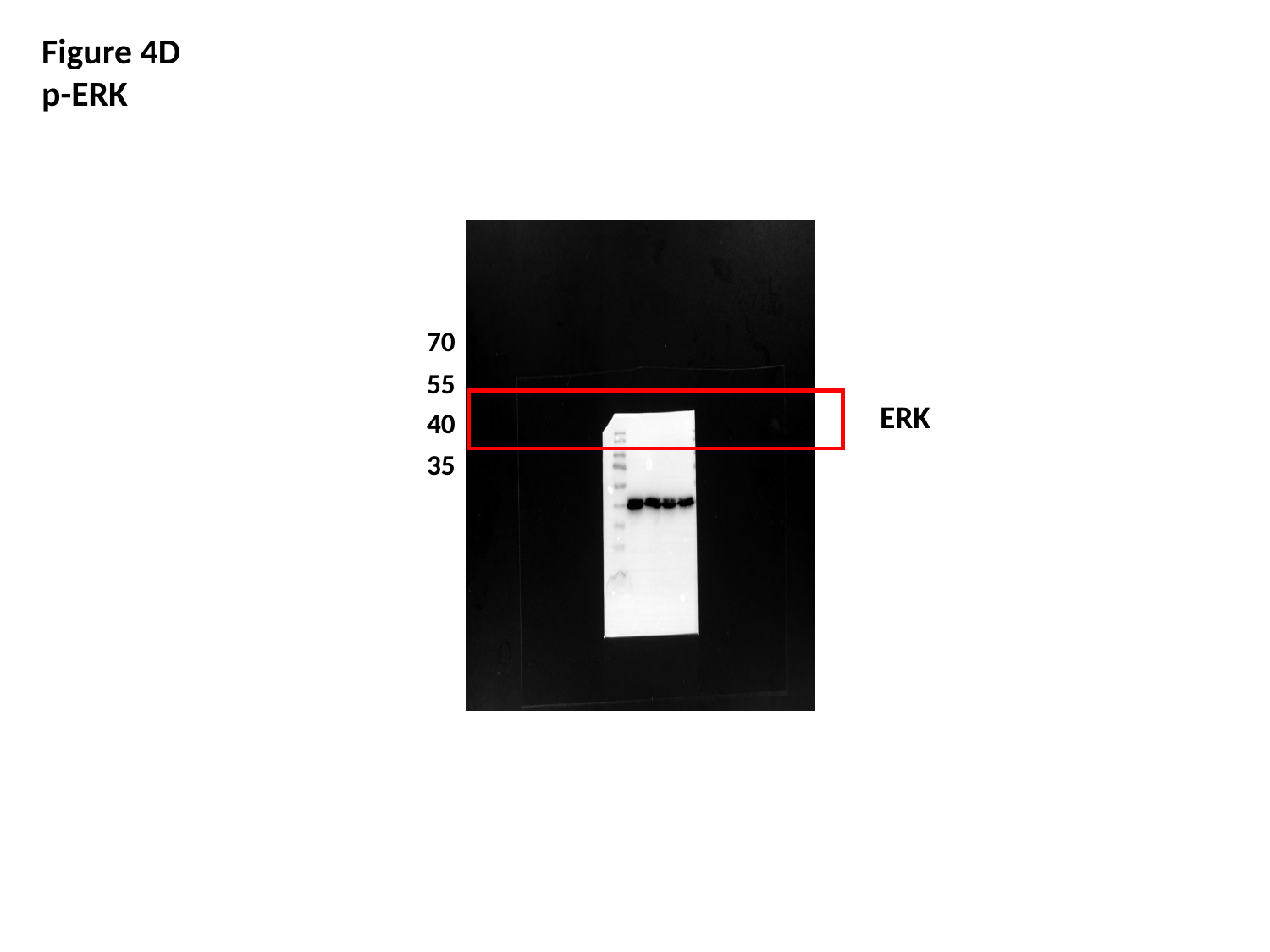

Figure 4D
p-ERK
70
55
ERK
40
35

## Slide 8
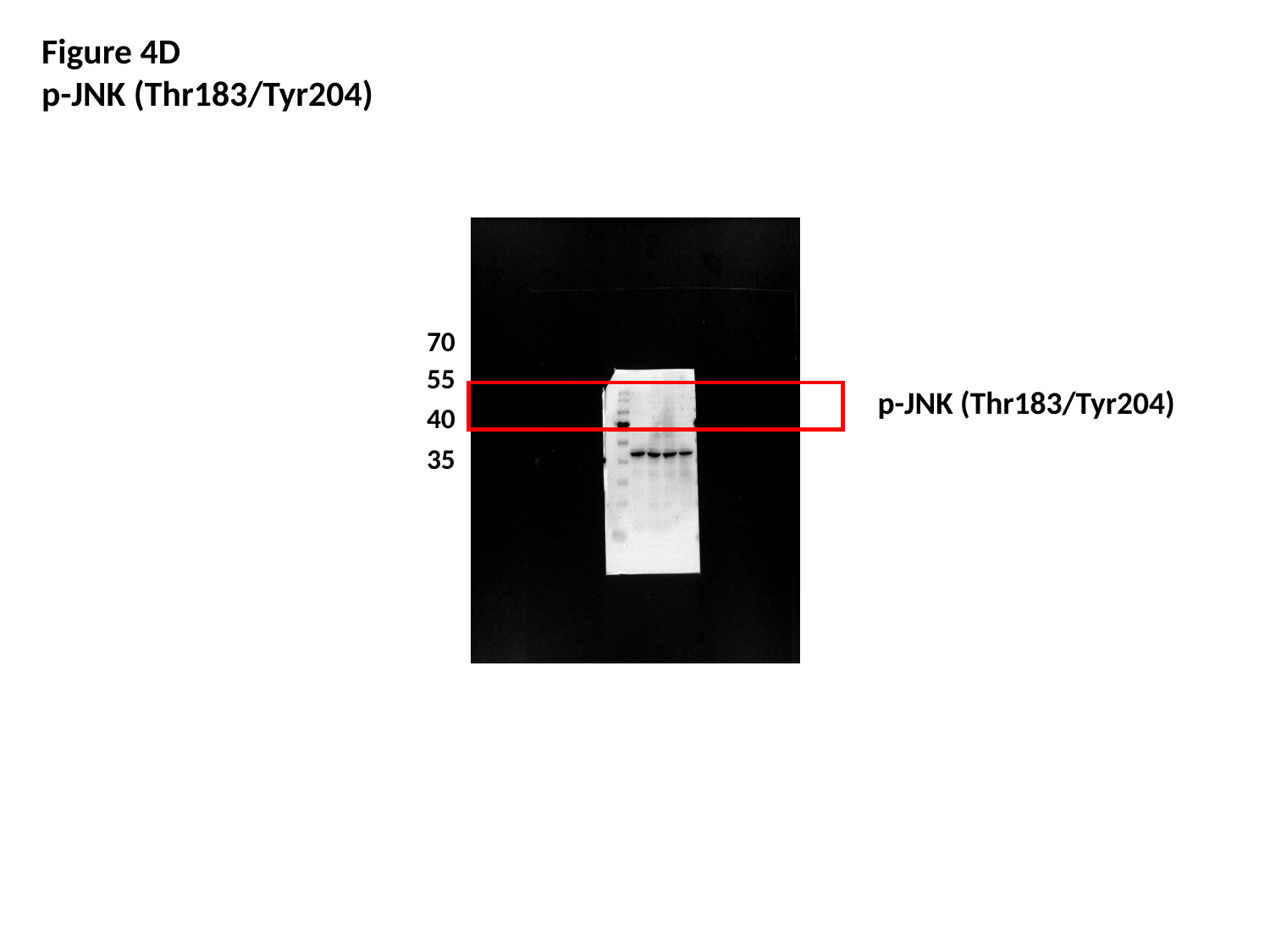

Figure 4D
p-JNK (Thr183/Tyr204)
70
55
p-JNK (Thr183/Tyr204)
40
35

## Slide 9
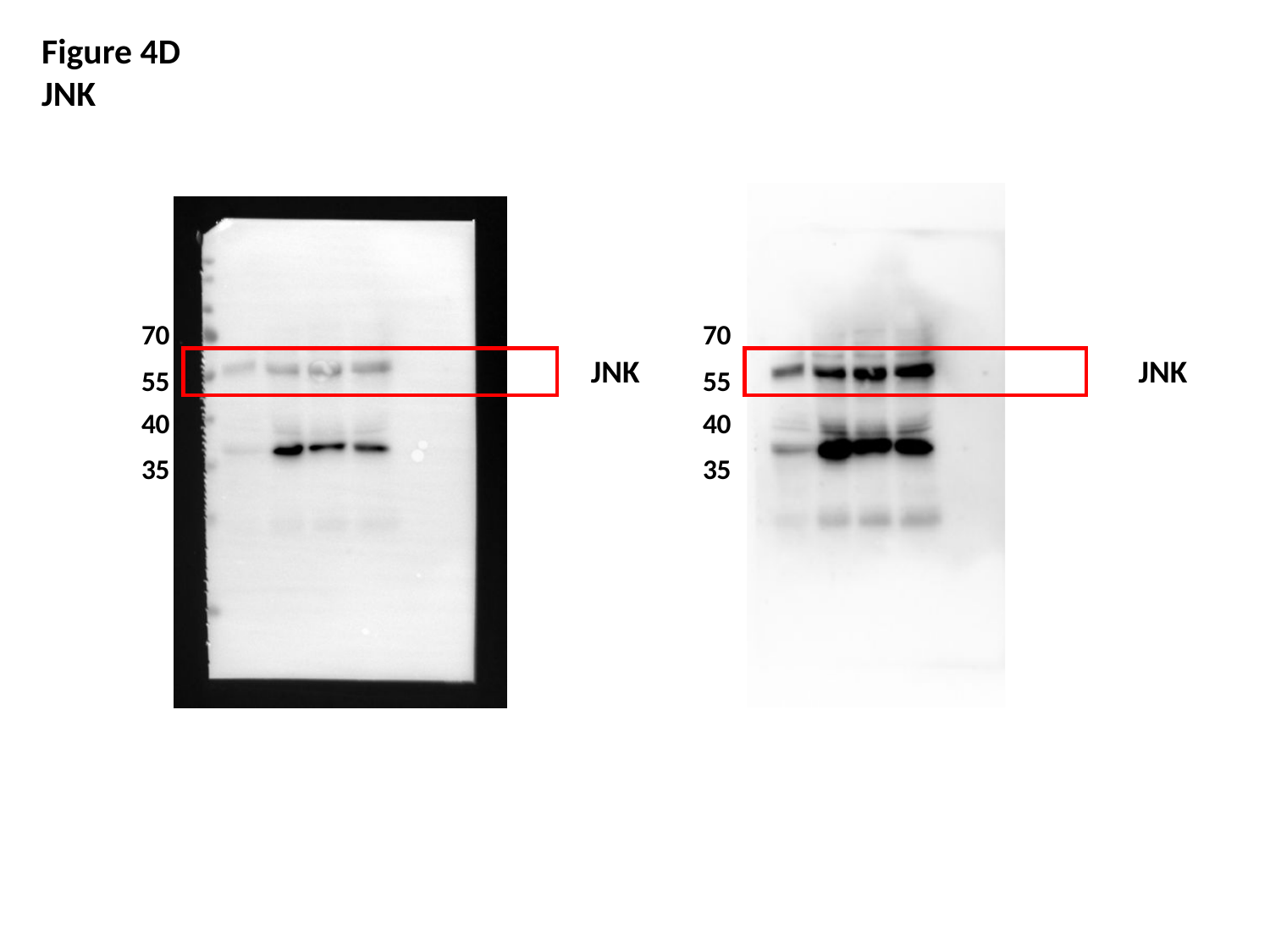

Figure 4D
JNK
70
70
JNK
JNK
55
55
40
40
35
35

## Slide 10
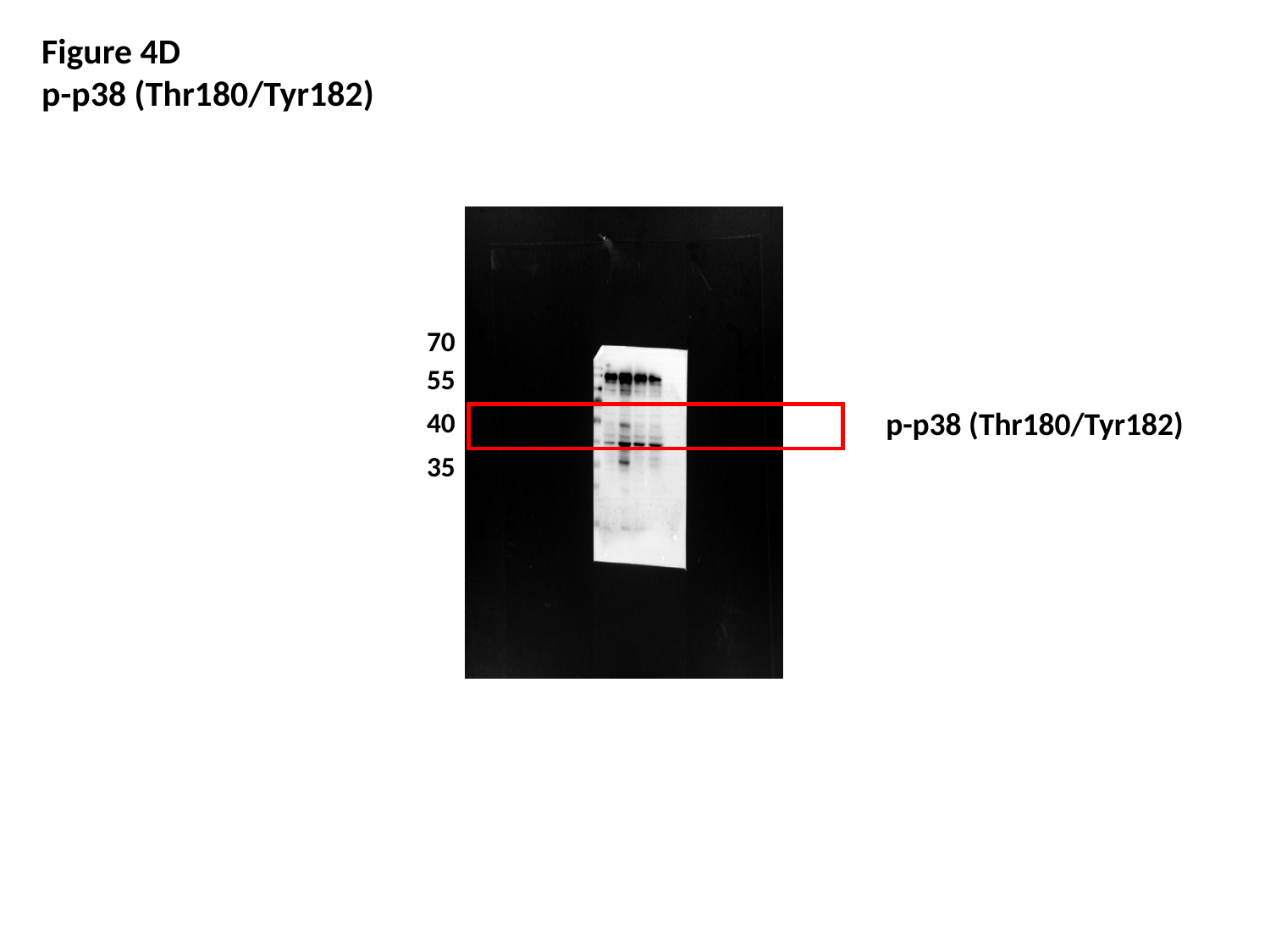

Figure 4D
p-p38 (Thr180/Tyr182)
70
55
p-p38 (Thr180/Tyr182)
40
35

## Slide 11
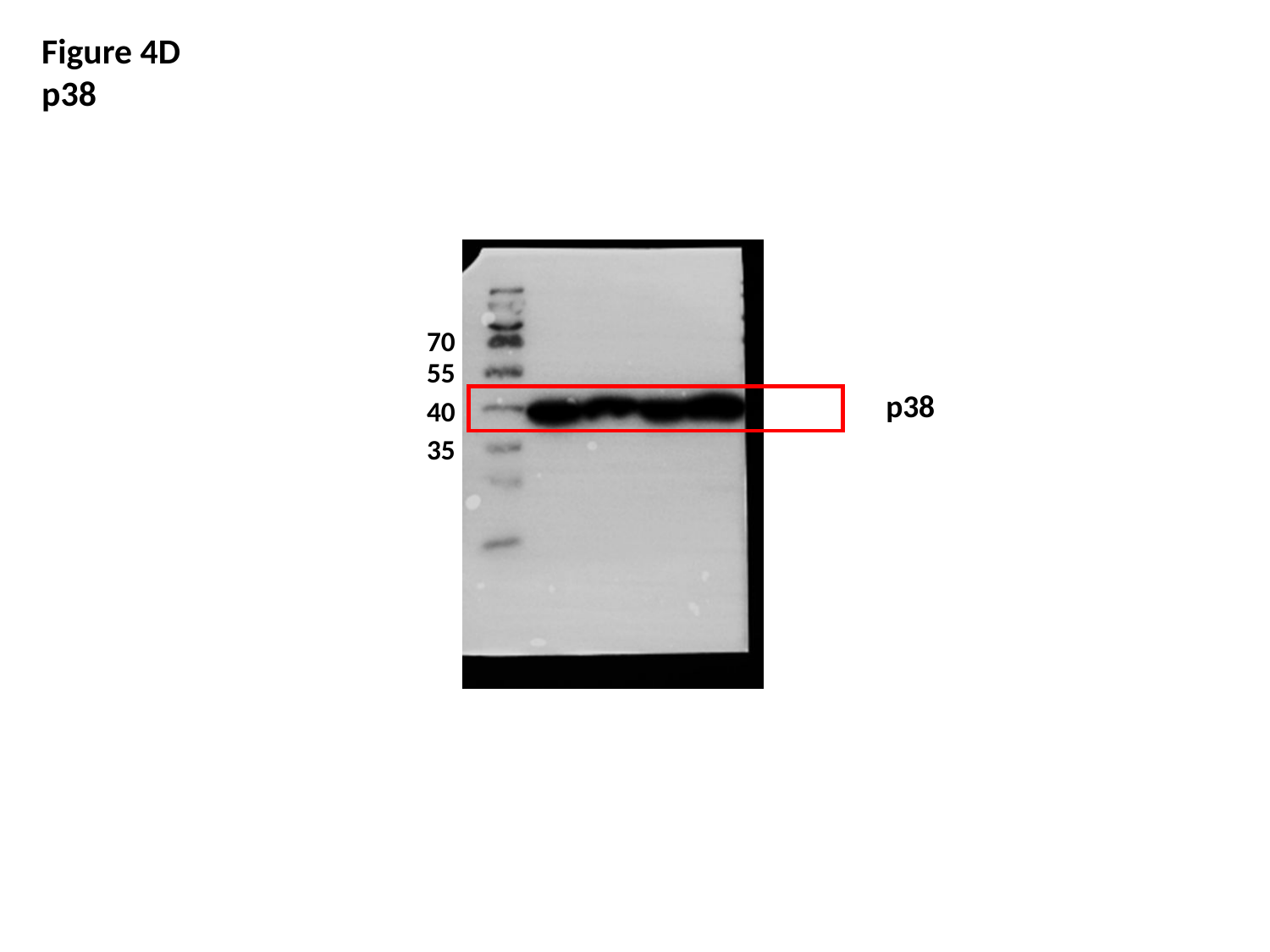

Figure 4D
p38
70
55
p38
40
35

## Slide 12
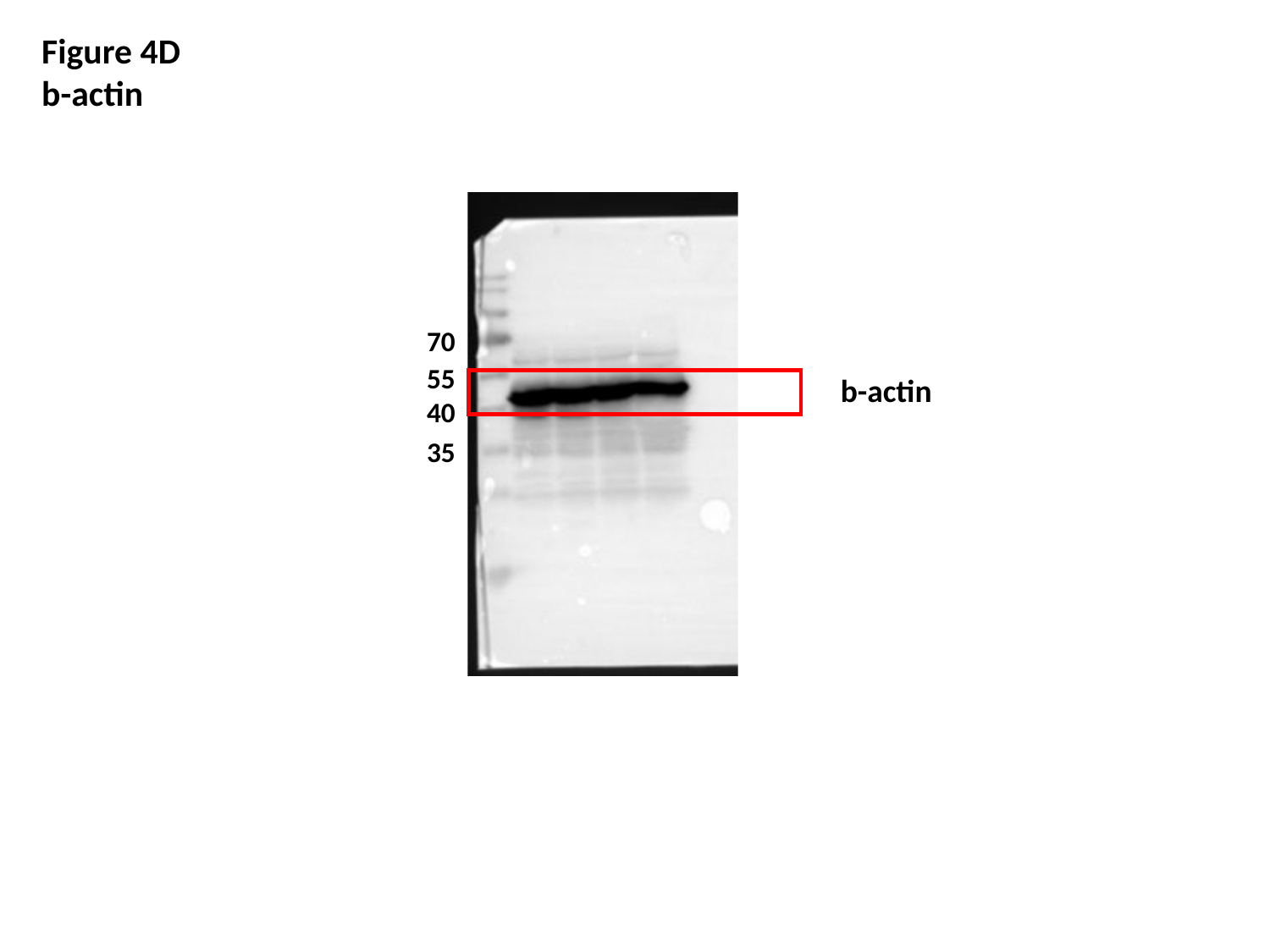

Figure 4D
b-actin
70
55
b-actin
40
35

## Slide 13
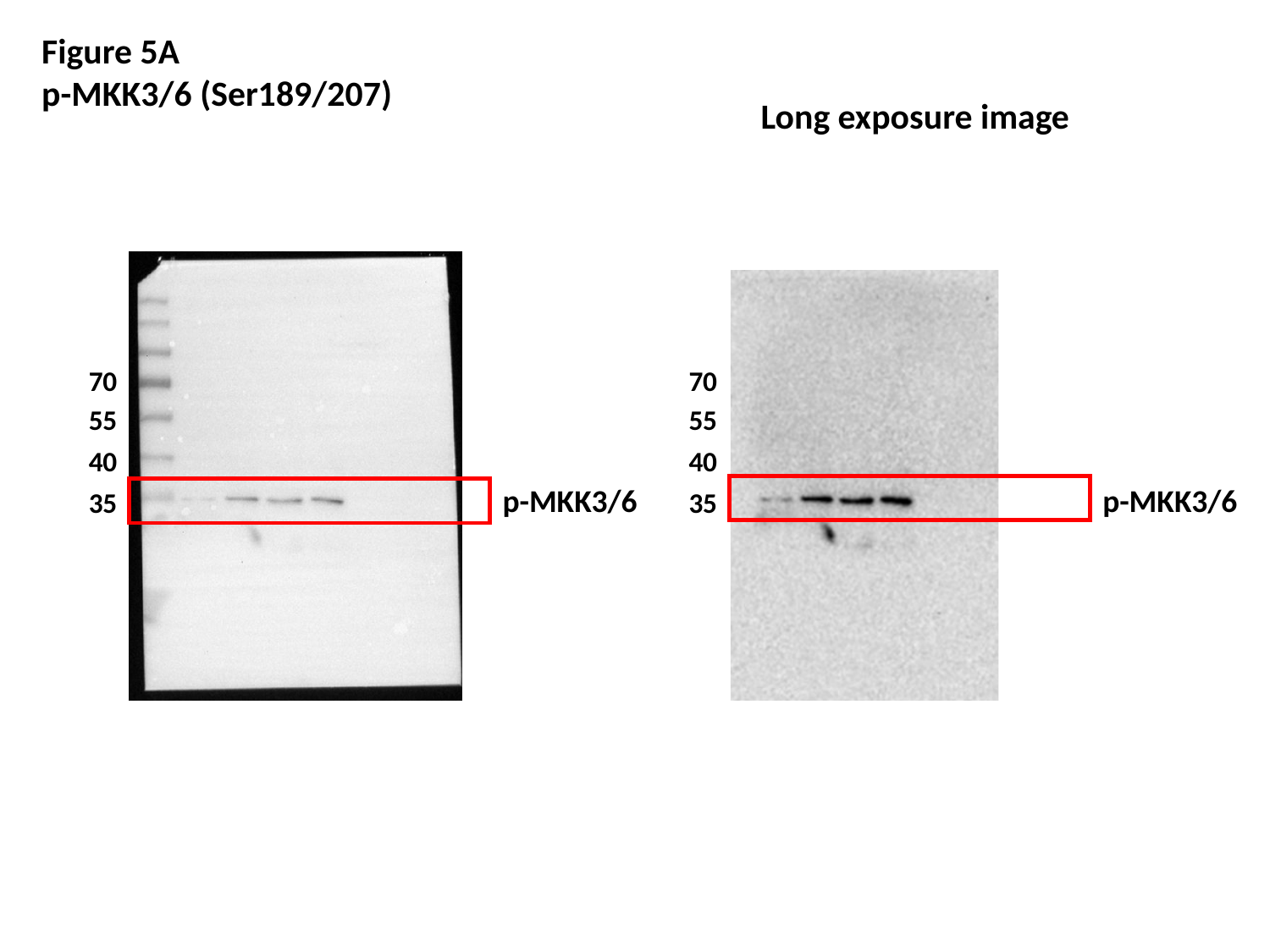

Figure 5A
p-MKK3/6 (Ser189/207)
Long exposure image
70
70
55
55
40
40
p-MKK3/6
p-MKK3/6
35
35

## Slide 14
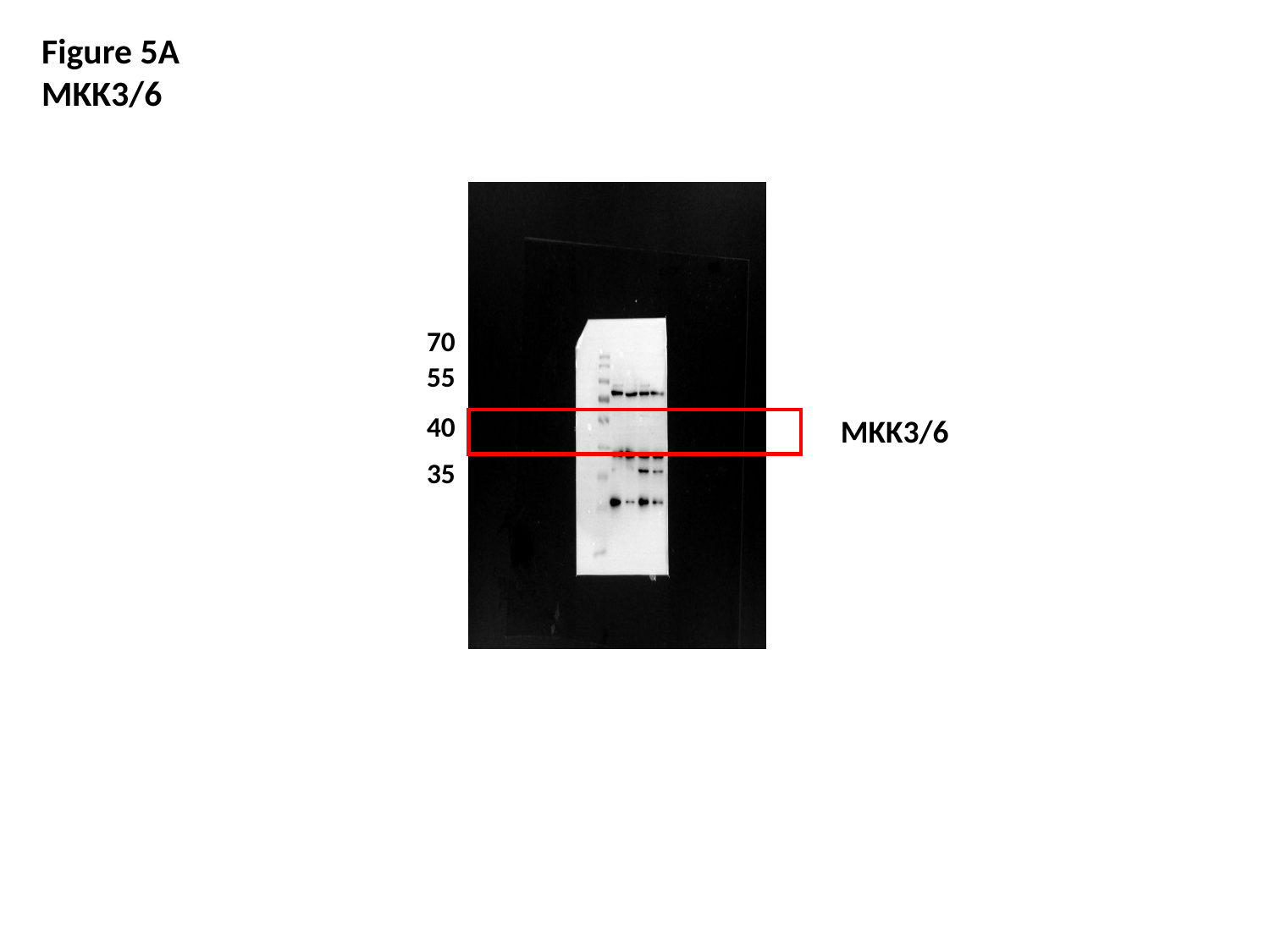

Figure 5A
MKK3/6
70
55
40
MKK3/6
35

## Slide 15
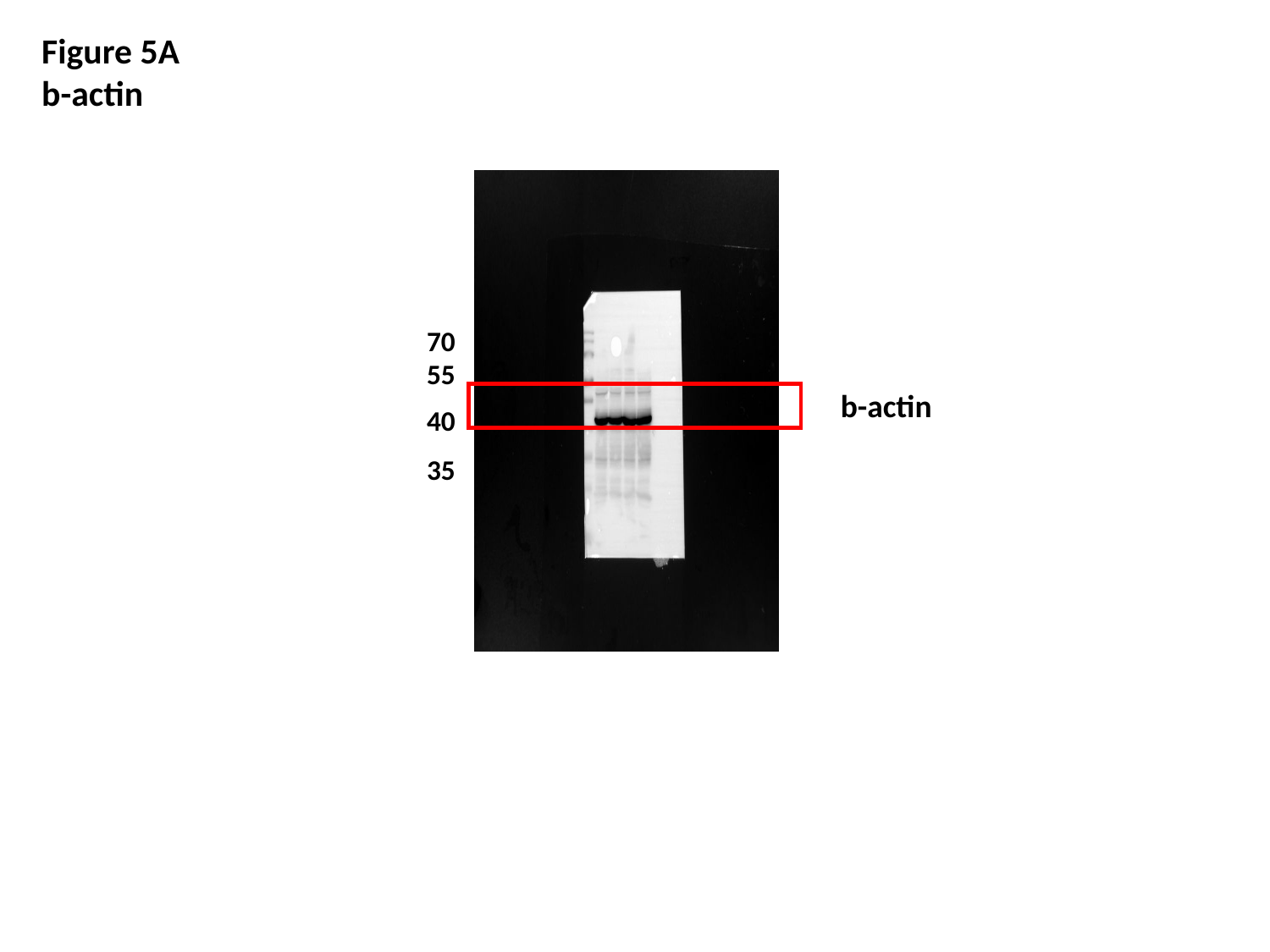

Figure 5A
b-actin
70
55
b-actin
40
35

## Slide 16
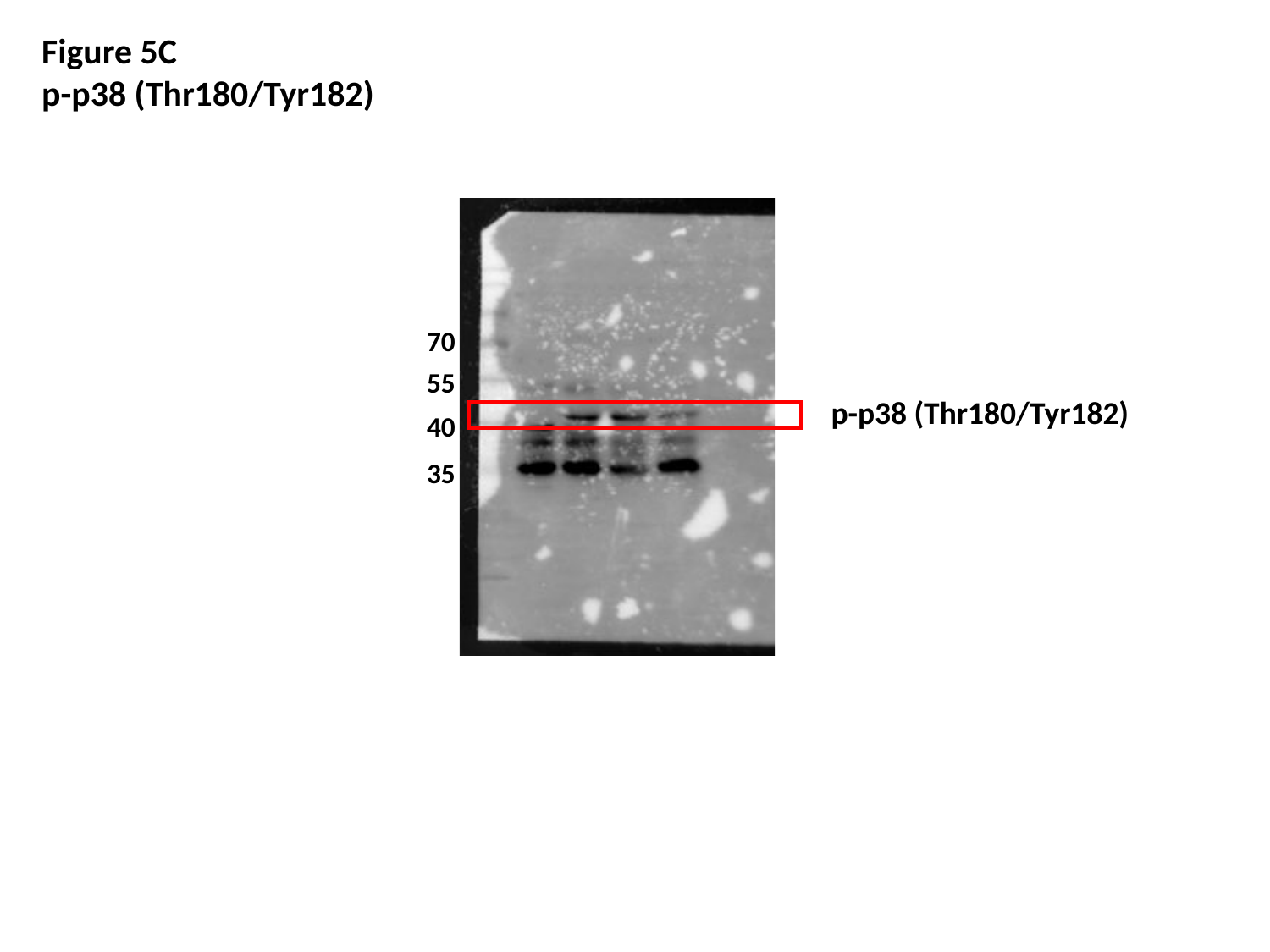

Figure 5C
p-p38 (Thr180/Tyr182)
70
55
p-p38 (Thr180/Tyr182)
40
35

## Slide 17
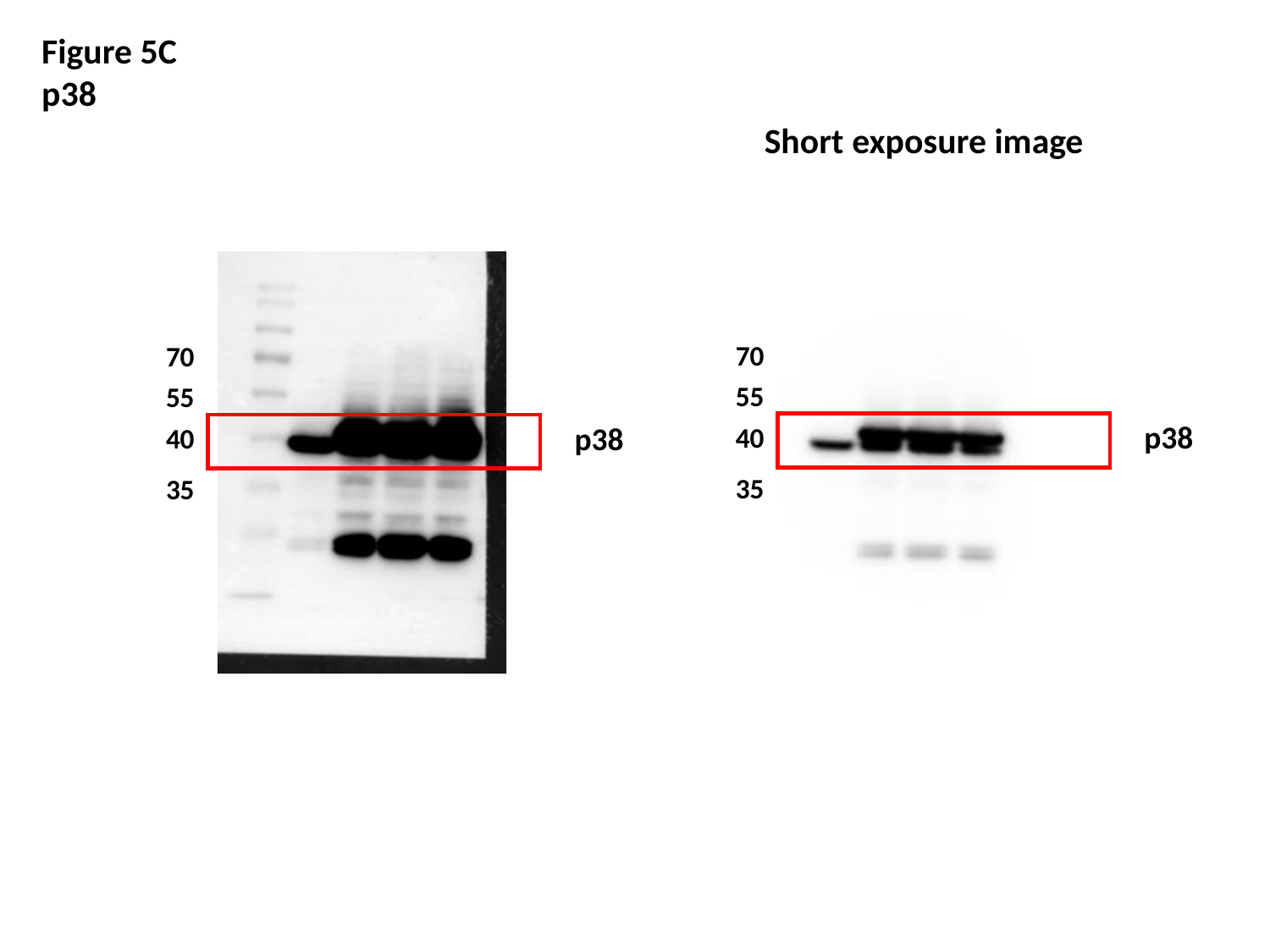

Figure 5C
p38
Short exposure image
70
70
55
55
p38
p38
40
40
35
35

## Slide 18
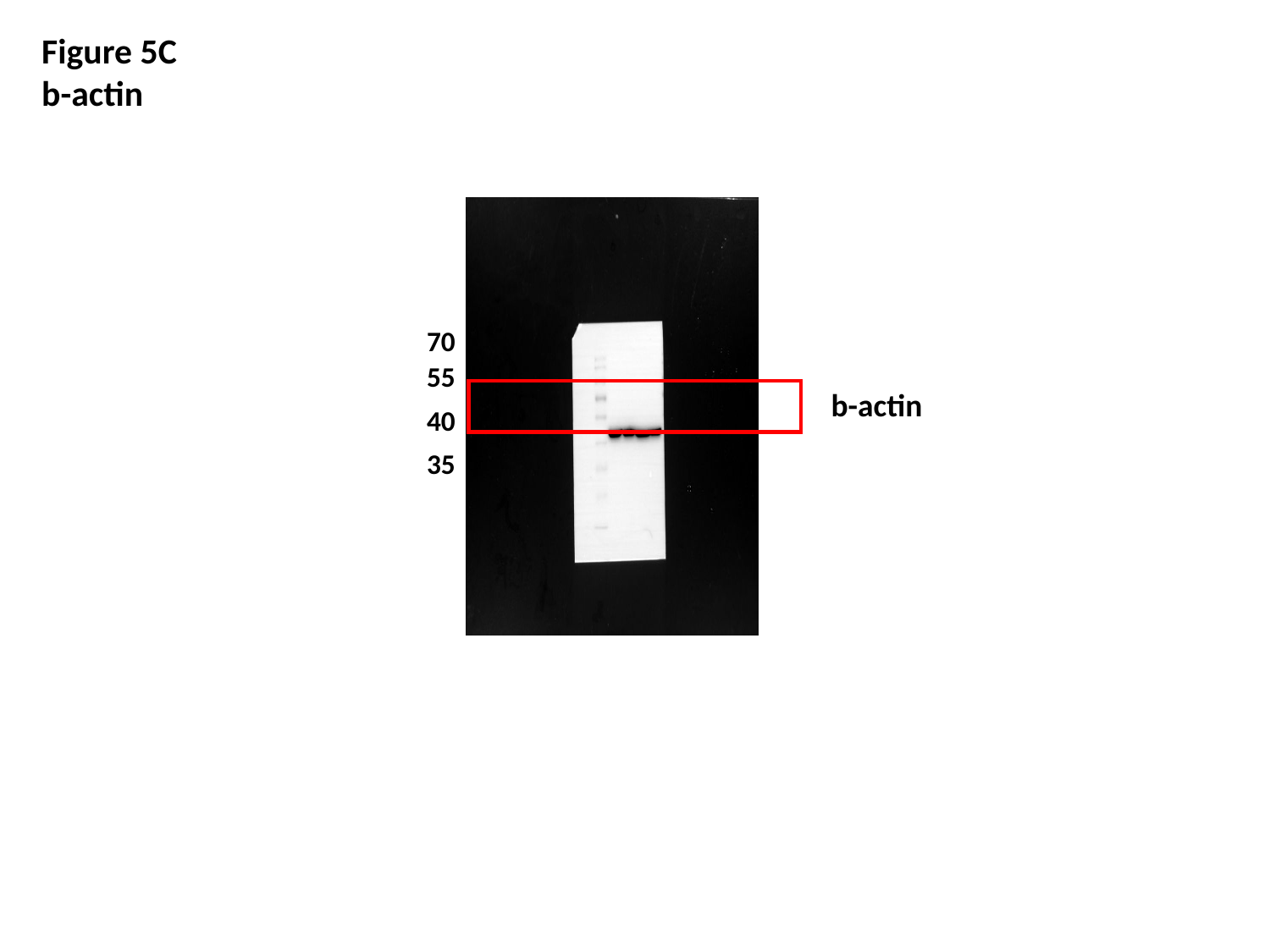

Figure 5C
b-actin
70
55
b-actin
40
35
